# Estradiol enhances B cell humoral immune responses against genital herpes simplex virus type 2 in mice through an IL-17 dependant pathway

**DOI:** 10.3389/fimmu.2025.1691163

**Published:** 2025-12-01

**Authors:** M. Firoz Mian, Ramtin Ghasemi, Puja Bagri, Joshua J. C. McGrath, Danya Thayaparan, Maysa Niazy, Denis P. Snider, Charu Kaushic

**Affiliations:** 1 Department of Medicine, McMaster University, Hamilton, ON, Canada; 2 McMaster Immunology Research Centre, Michael G. DeGroote Center for Learning and Discovery, McMaster University, Hamilton, ON, Canada

**Keywords:** estradiol (E2), herpes simplex virus type 2 (HSV-2), TK-HSV-2, intranasal immunization, memory B cells (MBC), plasma cells, IL-17

## Abstract

**Introduction:**

Estradiol has been shown to enhance anti-viral immunity and protect against HSV-2 infection. Previously, we reported that intranasal immunization with attenuated HSV-2 (TK-) in the presence of estradiol (E2) showed enhanced Th17 responses that led to increased anti-viral Th1 immunity in HSV-2 post-challenge. Whether enhanced Th17 cells also lead to improved B cell antibody responses against HSV-2 challenge in immunized mice in the presence of E2 was not examined and is the focus of the current study.

**Methods:**

Ovariectomized (OVX) C57BL/6 or IL-17 knockout (IL-17-/-) mice were implanted with 17β-estradiol (E2) or placebo pellets subcutaneously. Two weeks later, mice were immunized intranasally with a single dose of 10^4^ pfu of TK- HSV-2 and 4- or 6-weeks later, blood sera and vaginal washes were collected to measure IgG total and subtypes by ELISA. Mice were challenged intravaginally with 10^4^ pfu of WT HSV-2 4–6 weeks post-immunization, and vaginal washes were collected daily until euthanized at day 5 post-challenge to determine viral titers and protection. Mononuclear cells isolated from vaginal tract, spleen, nasal associated lymphoid tissue (NALT), cervical lymph nodes (cLN) and iliac lymph nodes (iLN) tissues were analyzed by flow cytometry for plasma and memory B cell phenotypes.

**Results:**

E2-treated WT OVX immunized mice after intravaginal HSV-2 challenge showed significantly increased HSV-2-specific IgG2b and IgG2c antibodies in serum and vaginal secretions compared to placebo mice and enhanced B220-CD138+ IgG2c+ plasma cells within the nasal mucosa and vaginal tract 6-weeks after immunization. Furthermore, E2 treatment enhanced the subsets of CD19+ IgD- memory B cells 4-weeks post immunization within the iLN and spleen. Notably, E2-induced increased B cell antibody responses conferred greater protection from HSV-2 challenge compared to placebo mice as evidenced by 2–3 logs decreased viral titers in the vaginal tract and 20% mice with genital pathology compared to 80% in placebo group, indicating better protection in E2-treated mice. Importantly, E2-mediated enhanced plasma and B cell antibody responses observed in WT mice were abrogated in IL-17-/- mice that led to 2–3 logs higher viral titers that were equivalent in WT placebo- and IL-17-/- mice and no difference in protection.

**Conclusion:**

This study provides novel evidence that part of the E2-induced enhanced anti-viral response is mediated by increased B cell antibody responses that requires IL-17. Thus, E2 could be exploited in developing an effective mucosal vaccine driving B cells through intranasal immunization to elicit stronger HSV-2-specific antibody responses in the female genital tract.

## 1 Introduction

Understanding the immune mechanisms that protect the female genital tract (FGT) against viral sexually transmitted infections (STI) in animal models can contribute to better design of vaccines to protect women (1). Genital herpes simplex virus type 2 (HSV-2) remains one of the most prevalent STIs worldwide with the highest prevalence in Sub-Saharan Africa (2). Globally, there were 25.6 million people aged 15–49 years detected with new HSV-2 infections, and 519.5 million (13.3%) with existing HSV-2 infections (3). HSV-2 causes primary infections in the genital mucosal epithelial layers and develops latent infections in sacral ganglia that re-activates intermittently leading to further transmission (4). Despite significant efforts to understand the pathogenesis and immunity to this virus, an effective vaccine against HSV-2 has not been developed yet.

An effective vaccine against HSV-2 should induce sterile immunity in the FGT in order to prevent the virus from establishing latency (5). Attempts to develop an antibody-mediated vaccine against HSV-2 began as early as the 1980s. Early studies on vaccine candidates focused primarily on eliciting antibody-mediated protection through systemic routes of immunization and demonstrated limited success in clinical trials (6, 7). Although each candidate vaccine induced systemic immune responses, they were unsuccessful in providing protection against HSV-2 in the genital tract (8). In order to generate local anti-viral immunity in the genital mucosa, a number of studies have highlighted the importance of mucosal immunization (1, 9–12). Earlier experimental studies have demonstrated the important role of IFN-γ secreting CD4+ T cells in protecting against WT HSV-2 following intravaginal (ivag) immunization (4, 13–15). Comparable strong mucosal protection was observed with both ivag and intranasal (i.n.) immunization, though i.n. immunization was found as a more practical and less intrusive vaccination strategy (16). Antibody responses in these models were found to be important for long term protection against re-infection (17). Therefore, a vaccine strategy that is capable of inducing both cellular and humoral immunity seems the best option for optimal protection.

Female sex hormones, estradiol (E2) and progesterone (P4), are known to regulate the susceptibility and immune responses to STIs including HSV-2 in the reproductive tract (18–21). A number of clinical and experimental studies have revealed that P4 appears to enhance the susceptibility to HIV-1 and HSV-2 (22–24), while E2 on the other hand, provides robust protection against lethal HSV-2 challenge in experimental mouse models (10, 12, 23, 25). We have demonstrated that i.n. immunization of E2-treated mice activates dendritic cells (DCs) which program an enhanced Th-17 response and leads to the establishment of a CD4+ tissue-resident memory cell (T_RM_) IFN-γ response in the FGT to provide enhanced protection against lethal challenge with HSV-2 (10–12). Specifically, we found that both IL-17- and IFN-γ secreting CD4+ T cells were induced one-week post-i.n. immunization within the cervical lymph nodes (cLN) and nasal associated lymphoid tissue (NALT), which was enhanced by E2, indicating the effect of E2 is not limited to the FGT in OVX mice. We and others have revealed that E2 treatment enhances HSV-2-specific serum and vaginal IgG levels, which lead to better protection in mice (24, 26). The role of E2 on B cell and antibody responses to HSV-2 was not examined in these studies.

Under normal physiological conditions, B cells are mostly absent within the mouse FGT and only infiltrate into the tissue in the presence of local inflammation, such as upon HSV-2 infection (17). Several studies have demonstrated that protection from WT HSV-2 in immunized mice is independent of B cell antibody responses (4, 13, 27, 28), while others revealed that immunized mice are protected from HSV-2 through B cell-dependent responses in the FGT (17, 29, 30). Furthermore, immunized B cell-deficient (μMT) mice had significantly higher viral titers of HSV-2 at 24-hours post-challenge with a 2-day delay in virus clearance compared to immunized WT mice, suggesting a role for B cells during early control of infection (28). Iijima et al. (4) showed that collective depletion of DCs and B cells, but not depletion of DCs or B cells alone, abrogated Th1-mediated protection from WT HSV-2 challenge in mice, suggesting that B cells primarily served as antigen-presenting cells (APCs). A few earlier studies performed in mouse model revealed a potential role for B cells and different immunoglobulins in the context of HSV-2 infection (31) indicating that the presence of HSV-2-specific IgG, but not secretory immunoglobulin A (IgA), within the vaginal secretions provides protection against HSV-2. In addition, affinity purified IgG, but not IgA, and adoptive transfer of serum IgG from immunized mice into the FGT of naive mice, reduced viral load after HSV-2 infection (9, 32). Notably, a recent mouse model study found IgG2b and IgG2c to be the primary IgG isotypes secreted in response to HSV-2 (30). Studies showed that i.n. immunization heavily relies on B cells since B cell-deficient (JHD/μMT) mice were not protected from subsequent WT HSV-2 challenge (29, 30). We have previously demonstrated that vaginal IgG correlated with decreased genital pathology in intranasally TK- immunized mice challenged with HSV-2 that were treated with E2, but not subcutaneously immunized or P4-treated mice. This revealed that protection following i.n. immunization is mediated by the secreted antibodies present in circulation rather than the B cells themselves. Despite a number of studies conducted on B cells in the context of HSV-2 immunization and challenge, humoral immune responses mediated by B cells remain unclear.

A cutting-edge mouse model study (33) has demonstrated that Th17 cells and their signature cytokines trigger B cell proliferation, promote the formation of the germinal center (GC) and isotype class switch to IgG1, IgG2a, IgG2b, IgG2c and IgG3. We have demonstrated that E2 treatment enhances both IFN-γ and IL-17 secreting CD4 T cells and provides complete protection against WT HSV-2 in OVX mice (10–12). We therefore hypothesized that in mice immunized intranasally with TK- following E2 treatment, IL-17 may play an important role to enhance B cell antibody responses against HSV-2 in the vaginal tract, thus contributing to the superior protection observed in E2-treated mice. In this study, we report that i.n. immunization in the presence of E2 leads to the enrichment of various memory B cell (MBC) subsets within peripheral secondary lymphoid tissues and antibody-secreting plasma cells within the nasal effector sites. Upon subsequent WT HSV-2 ivag challenge, this enriched MBC response leads to a more rapid and robust protective response within the FGT by establishing increased levels of HSV-2-specific IgGs within the vaginal lumen, leading to superior protection in an IL-17 dependent manner.

## 2 Materials and methods

### 2.1 Mice

Six to eight week old female C57BL/6 mice were purchased from Charles River Laboratories (Saint-Constant, Quebec, Canada). IL-17 knockout (IL-17-/-) mice were bred and maintained internally at the Central Animal Facility, McMaster University. Animals were maintained under specific pathogen-free and standard temperature-controlled conditions that followed a 12-hour light/dark cycle at the Central Animal Facility, McMaster University. Mice were allowed one week acclimatization after arrival prior to experimental use. All mouse experiments performed in this study were approved by and in compliance with the Animal Research Ethics Board (AREB) at McMaster University.

### 2.2 Ovariectomy

The endogenous source of sex hormones was removed by ovariectomies (OVX) as previously described (34). Briefly, mice were given analgesic (Carprofen 0.5mg/mL) through subcutaneous injection 30 minutes prior to the surgery. Mice were then anaesthetized intraperitoneally with injectable anesthetics (100 mg of ketamine/kg and 10 mg of xylazine/mL). Ovaries are located proximally near the hind limbs of the murine abdomen and were excised through incisions. The incisions were closed by suturing abdominal muscles and then the skin was surgically clipped. The animals were monitored two weeks post-surgery and allowed to recover before any experiments were conducted.

### 2.3 Hormonal pellet implantation

21-day release E2 pellet (476 ng/mouse/day) was implanted subcutaneously (Innovative Research of America, Sarasota, Florida, USA) using a previously published protocol (24). The concentration of serum E2 resulting from the pellets has been demonstrated to correspond to approximately 15pmol/l which is in the range of that measured during the estrus cycle in mice (35). Briefly, OVX mice were put under inhalation anesthesia Isoflurane (Bimeda-MTC, Cambridge, ON) and a small incision was made in the outer skin in the scruff region and the pellet was placed under the skin. The skin was closed with surgical clips and mice were monitored for 14 days for full recovery.

### 2.4 TK- HSV-2 intranasal immunization

Two weeks after E2 or placebo pellet implantation, mice were anaesthetized using gaseous anesthetic isoflurane. Mice were then immunized with 5 μL of thymidine kinase-deficient (TK-) HSV-2–10^4^ plaque forming unit (PFU) per mouse into each nare with a micropipette.

### 2.5 WT HSV-2 intravaginal challenge

Four to 6 six weeks following immunization, mice were anaesthetized using injectable anesthetics (150 mg of ketamine/kg + 10 mg of xylazine/kg) given interperitoneally. Mice were then inoculated intravaginally with 10^4^ PFU of WT HSV-2 strain 333 in 10 μl volume per mouse and laid on their back on a heating pad for about one hour to allow the virus to infect. Mice were monitored daily for genital pathology and survival.

### 2.6 Collection of vaginal secretions to assess viral shedding

Vaginal washes were collected daily for 5 consecutive days after ivag WT HSV-2 challenge by pipetting 30 μL of PBS twice consecutively in and out of the vagina 5–6 times to give a total volume of approximately 60 μL, centrifuged at 800g for 10 minutes and stored at -80°C.

### 2.7 Serum collection

Serum samples were collected at 4 or 6 weeks post-immunization and day 5 post-HSV-2 challenge. Briefly, animals were anesthetized using gaseous anesthetic Isoflurane (Bimeda-MTC, Cambridge, ON). For post-i.n. immunization, blood samples were collected by retro-orbital bleeding using the micro-capillary tubes. For post-ivag challenge, blood samples were collected by cardiac puncture. In either case, the samples were allowed to clot at room temperature for 30 minutes. The clotted blood was then centrifuged at 8000 x g force for 10 minutes and the serum was collected and stored at -80°C.

### 2.8 Genital pathology

Genital pathology following challenge with WT HSV-2 was monitored daily until the end of the experiment and was scored on a five-point scale as described before (23): 0, no infection; 1, slight redness of external vagina; 2, swelling and redness of external vagina; 3, severe swelling and redness of both vagina and surrounding tissue and hair loss in genital area; 4, genital ulceration with severe redness, swelling, and hair loss of genital and surrounding tissue; 5, severe genital ulceration extending to surrounding tissue and hind limb paralysis. Animals were euthanized when they reached stage 4 to 5 as per approved procedures of Animals Utilization Protocol.

### 2.9 Viral plaque assays

Plaque assays were used to quantify the viral replication in the vaginal epithelium, as described previously (23). The vaginal washes stored at -80 °C were thawed on ice, then diluted serially from 10^–2^ to 10^–7^ in serum-free alpha-MEM media. Each dilution was added onto a confluent monolayer of Vero cell cultures in 12-well plates. Plates were incubated at 37°C with 216 uL for 2 hours with intermittent swirling to facilitate viral entry evenly and then overlaid with 5% FBS containing alpha-MEM complete medium to prevent new attachment of remaining virus in the samples. After 48 hours, plates were stained with crystal violet and visualized on a light microscope to count the total plaques (each plaque formed in Vero cells determines a viral PFU). To obtain the total viral count in a lavage sample, the counted plaques were multiplied by the dilution factor and presented as PFU/ml.

### 2.10 HSV-2 specific antibody neutralization assay

An *in vitro* antibody neutralization assay was performed to evaluate the association between the viral loads and the level of antibody present in the serum and vaginal washes collected from E2 vs placebo treated immunized mice challenged with HSV-2. To perform this, Vero cells were seeded in 96-well tissue culture plate (25, 000 cells/well) to attain a confluent monolayer in 24 hours. In a set up plate (96-well U-bottom plate), 60 ul of serum and vaginal wash samples were added to 60 ul of serially diluted GFP-HSV-2 virus to obtain MOI 1.0, 0.5, 0.1 and 0.05 to optimize virus dose to see virus neutralization and incubated 1 hour at 37°C. Then carefully added 100 ul mixture from set up plate containing antibody and virus dilutions to replace the medium in corresponding wells of the Vero cells plate and top up with 100 ul of serum-free alpha-MEM medium. The plate was then incubated at 37°C for 2 hours with intermittent swirling to allow virus to infect Vero cells. Virus containing medium was removed and replaced with 200 ul of 5% FBS-containing alpha-MEM complete medium and incubated at 37°C for 24 hours and plate was read for GFP fluorescence in CYTATION 7 (Agilent BioTek Inc, Canada) fluorescence plate reader at wavelengths 395/509 nm excitation/emission to obtain GFP fluorescence images as well as counted GFP+ cells as indicative of HSV-2 virus. We determined MOI 0.5 as the optimum virus dilution and used for the antibody neutralization assay to assess all serum and vaginal samples.

### 2.11 Isolation of cells from different tissues

#### 2.11.1 Vaginal tract

The vaginal tract was collected and processed according to a standardized protocol previously described (36). Briefly, mice were euthanized by cervical dislocation according to our animal use protocol (AUP) approved by McMaster Animal Research Ethics Board and vaginal tract excised and collected in complete RPMI 1640 medium on ice. The vaginal tract was opened to expose the inner epithelial layer and cut into small pieces. Vaginal tissue pieces were digested in 15 mL of RPMI 1640 containing 0.00157 g/mL collagenase A (Roche Life Science, USA) at 37°C with vigorous shaking for 1 hour, followed by a second round of digestion. The suspension was processed by filtration through 40 μm filter. The resultant homogenous cellular suspension was spun down and the pellet was resuspended in 0.5 mL of FACS buffer.

#### 2.11.2 Nasopharynx-Associated Lymphoid Tissue

NALT tissues were isolated and processed, as described before (37). The tissue was isolated according to a previously published protocol (38). Briefly, animals were euthanized by cervical dislocation and the lower jaw, tongue and connective tissues were removed exposing the soft palate of the upper jaw. The palate was peeled back, revealing the pair of NALT structures that were positioned at the posterior of the hard palate. The palate was isolated using forceps and washed with 250 μL of RPMI-1640 media and resuspended in 1 mL of RPMI-1640 media in a 1.5 mL Eppendorf tube. Subsequently, the collected tissue was processed by placing tissue pieces between two frosted glass slides and grinding it to homogeneity. Finally, the resultant homogenous cellular suspension was spun down and the pellet was resuspended in 250 μL of FACS buffer.

#### 2.11.3 Nasal mucosa

Nasal mucosa was isolated and processed as described before (37). Briefly, animals were euthanized by cervical dislocation. After excising the NALT, the skull was dissected and nasal mucosa was obtained by resecting 1/3 of mucosa from nasal septum exposed by extending incision of nasal skin and bone below nares to inner cavity. Nasal mucosa was removed from the nasal septum mucosa and washed extensively with RPMI-1640 media. A single cell suspension was obtained by filtering the tissue pieces through a 40 μm filter. The resultant homogenous cellular suspension was spun down and the pellet was resuspended in 500 μL of FACS buffer.

#### 2.11.4 Lymph nodes and spleen

Spleen cells were isolated according to our previously published protocol (39, 40). Briefly, spleens were mechanically disrupted, placed on a 40 µm filter on a 6-well plate, centrifuged and red blood cells were lysed using ACK lysis buffer (0.15 M Ammonium chloride, 0.01M Potassium bicarbonate, 0.0001 M Disodium EDTA in 1 L distilled water (pH 7.2), our laboratory made). The remaining cells were washed and resuspended in RPMI 1640 medium supplemented with 10% FBS, 100 IU/ml penicillin, 100 g/ml streptomycin, 1% L-glutamine, 0.1% 2-mercaptoethanol, 1% nonessential amino acids, and 1 sodium pyruvate (Gibco Life Technologies, Burlington, ON, Canada).

Cervical lymph nodes draining the upper respiratory tract (URT) and iliac lymph nodes draining the FGT and vagina were collected and mechanically disrupted by placing them on 40 uM filters on 6-well plates in complete RPMI 1640 medium. Cell suspensions were then washed by centrifugation and the pellet was resuspended in FACS buffer.

Single-cell suspensions from different tissues were resuspended in 1 to 5 ml of RPMI medium. Finally, live mononuclear cells were counted by using Trypan Blue stain that stains only dead cells, and cells were processed for flow cytometry staining.

### 2.12 Flow cytometric analysis

The cells isolated from different tissues were differentially counted on a hemocytometer using Trypan Blue staining to exclude dead cells, fibroblasts or epithelial cells. Staining was performed at 1–2 x 10^6^ cells/sample, depending on the tissue from which cells were isolated. Cells were incubated with anti-CD16/CD32 antibodies in 2% FBS-PBS buffer for 15 minutes to block the Fc receptors and then incubated 30 minutes in dark on ice with fluorescently conjugated antibodies for extracellular markers CD45-PerCP Cy5.5, CD19-BB515, B220-PE-Cy5, CD138-BV650, CD73-PE, CD80-BV510 and PD-L2-PE-Dazzle594 (BD Bioscience, Canada) followed by two washes with FACS buffer. To exclude dead cells, cells were subjected to staining with fixable viability dye (eFluor 780) in PBS for 20 minutes followed by two washes. Cells were then fixed in Cytofix/Cytoperm solution (BD Biosciences, Canada) and permeabilized with Perm/Wash Buffer before staining with intracellular antibodies (IgG2b-PE-Cy7, IgG2c-BV711, IgD-BV785, IgG3-BV421) in Perm/Wash Buffer for 30 minutes, followed by two washes with Perm/Wash Buffer. Finally, cell pellets were resuspended in 250 μL of 2% PFA FACS buffer and ran on the BD Fortessa flow cytometry machine (BD Bioscience, Mississauga, ON). All antibodies were validated and titrated for optimal conditions before their application in the experiments. The accuracy of staining positive cut-offs was verified by fluorescence minus one (FMO) controls. Results were analyzed using FlowJo software (Tree Star, Ashland, USA).

### 2.13 Anti-HSV-2 IgG ELISA

Ninety-six well Nunc MaxiSorp ELISA plates were coated with UV-inactivated WT HSV-2 (10^4^ pfu equivalent per 100 μL) for virus-specific Ig measurements and incubated overnight at 4°C. The next day, plates were washed with PBS Tween 20 and blocked with 5% casein for 6 hours. Serum and vaginal samples underwent two-fold dilution from neat to 16,384 for serum and neat to 1024 for vaginal washes. Higher dilutions were tested (100-10,000 fold) for serum and lower dilutions of vaginal washes (neat-100 fold) were tested in antibody assays based on typical amount of antibodies detected in previous studies. The above mentioned serially diluted serum and vaginal secretion samples were plated in wells (100 μL per well) and incubated overnight. After washing with PBS Tween 20 washing buffer, BIOTIN conjugated anti-mouse IgG1, IgG2b, IgG2c, or IgG3 (Southern Biotech, Birmingham, AL, USA), were added to each well at 1:500 dilution, followed by washing. Subsequently, 100 μL of Streptavidin–peroxidase (1:2000 diluted in PBS) was added to each well. Next, the plate was developed by the addition of 100 μL TMB solution. Reactions were stopped with 1N H2SO4 and absorbance (450 nm) was measured in the BioTek SynergyH1 plate reader (Agilent, Fisher Scientific). The detection level for the in-house assays for IgG isotypes was based on controls that were run during optimization of the assay where normal serum from unimmunized mice was run in the same assay and the OD for that was used as the cut off baseline value. Endpoint titers was determined by calculating two times the mean background optical density value of non-immunized serum or vaginal secretion. The last dilution of experimental sample above this cut-off was considered to be the endpoint titer. Total IgG concentrations in serum and vaginal wash samples were measured using a commercial Mouse IgG total ELISA kit (Thermo Fisher Scientific, ON, Canada) following the instructions provided by the manufacturer. The detection limit or sensitivity of the kit is 1.56 ng/ml.

### 2.14 Statistical analysis

Statistical analysis and graphical representation were performed using Graph-Pad Prism 10.0.2 (GraphPad Software, San Diego, CA). The Mantel-Cox log rank test was used to calculate significant differences in survival. Data are expressed as means with standard errors of means (SEMs) error bars, typically derived from minimum of 3 independent experiments, with n=5–10 mice/group. Normality for all data was assessed using the Shapiro–Wilk test (default for small to moderate samples) and *p* > 0.05, indicating that the data did not significantly deviate from normality. In addition, we visually confirmed the distribution using Q–Q plots, which showed that the data points closely followed the theoretical normal distribution. Therefore, the assumption of normality was considered satisfied for subsequent parametric analyses using unpaired *t* test and two-way ANOVA. Statistical tests were selected based on the number and structure of variables being compared. A two-tailed *t* test was used to compare the means between two independent groups when evaluating the effect of a single factor, whereas a two-way ANOVA was applied for analyses involving two independent variables or multiple dependent measures, allowing assessment of both main effects and their interaction. Data were considered statistically significant if the P values were <0.05. Significant differences are indicated as *P<0.05, ** P<0.01, *** P<0.001 or, **** P<0.0001.

## 3 Results

### 3.1 Estradiol treatment enhances plasma B cell antibody responses in the vaginal tract and viral clearance in TK- immunized mice challenged intravaginally with wild-type HSV-2

B cells are normally absent in the FGT and only recruited following HSV-2 exposure (17). Although our previous studies have shown that in E2-treated intranasally immunized mice, vaginal IgG levels correlate with decreased pathology following HSV-2 challenge, the B cell populations and their origin was not examined (24). To identify the major B cell population involved in protection against WT HSV-2, we analyzed the phenotype of the B cells infiltrating the vaginal tract post-i.n. immunization followed by ivag HSV-2 challenge. Based on a recent detailed kinetic study starting from day 0 up to day 60 following ivag HSV-2 challenge of mice immunized for 5 weeks with TK- HSV-2 and demonstrated that MBCs were detected as early as day 1 post-challenge, reached the peak level at day 5 and gradually declined by day 9 and were down to baseline by day 60 post-challenge (17), we chose day 5 post-challenge to examine the difference between E2 treated versus placebo treated mice.

**Figure 1:**
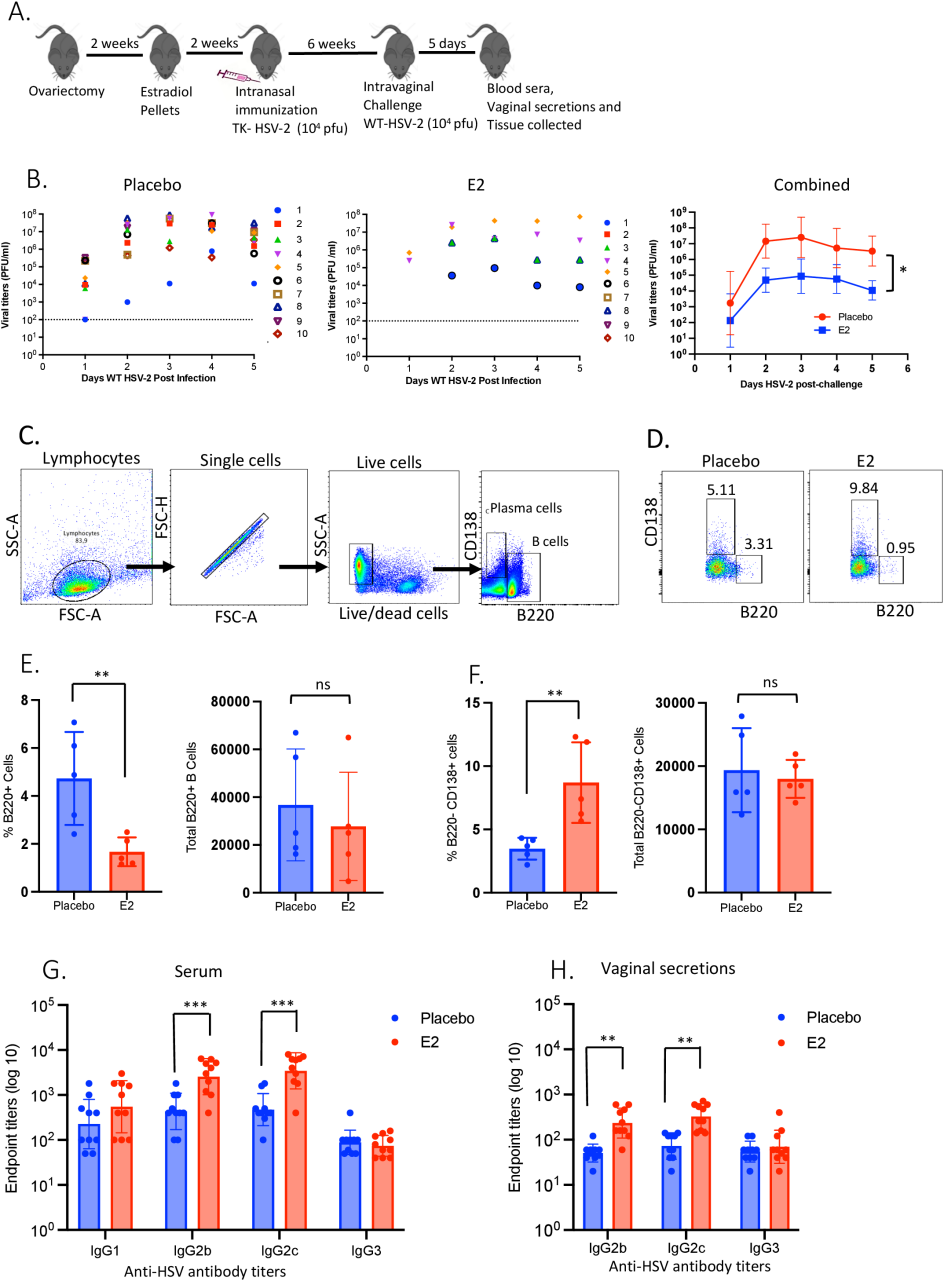
E2-treated TK- HSV-2 immunized mice following HSV-2 challenge show reduction in viral titers, increased proportion of plasma B cells as well as IgG2b and IgG2c secretions in serum and vaginal tract. WT C57BL/6 OVX mice were implanted with E2 or placebo pellets. Both groups were intranasally immunized with a single dose of TK- HSV-2 at 10^4^ pfu/mouse. Six weeks later, both groups were challenged ivag with WT HSV-2 at 10^4^ pfu/mouse. Vaginal washes were collected daily up to day 5 post-challenge for viral titers, and vaginal tissues were collected at day 5 post-challenge, pooled, processed, and mononuclear cells analyzed by flow cytometry. Serum samples and vaginal washes were collected at day 5 post-challenge to assess HSV-2-specific IgG1, IgG2b, IgG2c, IgG3 by ELISA and endpoint titers were determined. **(A)** Experimental design; **(B)** Vaginal washes collected daily for 5 days post-challenge were assessed for viral titers as depicted in separate panels for E2 and placebo groups, with the right-most graph showing the combined viral titers to compare between E2 and placebo treatments; **(C)** Gating strategy for B and plasma cells; **(D)** Representative dot plots showing frequencies of B cells (B220+) and plasma cells (B220-CD138+) gated from total live lymphocytes; **(E)** Bar graphs displaying percentages and total number of B220+ B cells were compared between placebo and E2-treated mice; **(F)** Percentages and the total numbers of B220-CD138+ plasma cells after HSV-2 challenge in the vaginal tract were compared between placebo and E2-treated mice; G-H) HSV-2-specific IgG1, IgG2b, IgG2c and IgG3 antibody titers at day 5 post-challenge as measured by ELISA in **(G)** serum samples, and H) vaginal secretions. Viral titers **(B)**, serum **(G)** and vaginal secretions **(H)** data shown are pooled from two independent experiments (n=10). Flow cytometry data **(E, F)** shown is a representative of one experiment from three independent experiments showing similar results, the bars indicate mean ± SEM (n=5 mice/group). Data for panels **(B, E, F)** were analyzed by the unpaired, two-tailed *t* test, and **(G, H)** data were analyzed by two-way ANOVA *P <0.05; **P <0.01; ***P<0.001.

As shown in the experimental outline in **Figure 1A**, WT C57BL/6 OVX mice were implanted with E2 or placebo pellets. Two weeks later, both groups were immunized intranasally with TK- HSV-2, and challenged intravaginally with WT HSV-2 six weeks later. Vaginal washes were collected daily and assessed to determine viral titers. Animals were sacrificed 5 days post-challenge and blood serum was collected. Mononuclear cells were isolated from vaginal tissue and analyzed by flow cytometry for subset of B cells.

We first assessed viral shedding in E2 treatment as compared to placebo controls. E2-treated mice, immunized intranasally with TK-HSV-2 and challenged intravaginally with WT HSV-2 6 weeks later, displayed significantly decreased viral shedding compared to placebo control mice, similar to what we have reported before (**Figure 1B**). Next, we examined the B cell responses elicited following i.n. immunization and ivag challenge and evaluated plasma B cells as the source of vaginal HSV-2-specific antibodies. Plasma cells were identified by the lack of expression of B220 and the presence of CD138+ following HSV-2 exposure (**Table 1**). **Figure 1C** demonstrates the gating strategy to identify B cells (B220+) and plasma B cells (B220-CD138+) as gated from total live lymphocytes. Notably, flow cytometry analyses of vaginal tract cells showed significant depletion of the proportion of B220+ cells (**Figures 1D, E**), while the percentages of B220-CD138+ plasma cells were significantly enhanced in E2-treated mice (**Figures 1D, F**) compared to placebo controls. However, we did not see any pronounced differences in absolute counts of B220+ or B220- CD138+ plasma cells between E2 and placebo treatments, indicating an overall enrichment of plasma cells. Assessment of serum and vaginal antibodies demonstrated that E2-treated mice had significantly elevated levels of HSV-2-specific IgG2b and IgG2c both in serum and vaginal secretions (**Figures 1G, H**), which coincided with decreased viral titers. In addition, E2 treated WT immunized mice challenged with HSV-2 showed significantly increased level of HSV-2-specific total IgG both in serum and vaginal washes comparing to placebo controls (**Supplementary Figure S1A**). To substantiate the *in vivo* findings that demonstrates an inverse association between the antibody levels and viral titers, we performed an *in vitro* neutralization assay examining the serum and vaginal wash samples in Vero cells using a GFP-HSV-2 virus. To this end, serum and vaginal wash samples were incubated with GFP-HSV-2 virus for an hour and then added these to confluent monolayers of Vero cells in 96-well plate to allow virus to infect cells. The plate was then read for GFP fluorescence in CYTATION 7 plate reader. Importantly, serum and vaginal washes collected from E2 treated mice demonstrated robust inhibition of virus infection as displayed by significant reduction in GFP+ Vero cells compared to placebo samples (**Supplementary Figure S2**). This *in vitro* data strongly corroborate the *in vivo* findings and re-confirmed that the E2-mediated reduction in viral loads in immunized mice is directly due to antibody-mediated virus neutralization. Together, these results demonstrate that i.n. immunization in the presence of E2 significantly reduced viral shedding post-ivag challenge, which is likely due to increased plasma cells in the vaginal tract and increased anti-HSV IgG2b and IgG2c antibodies in the vaginal tract and serum.

**Table 1:** Characteristics of B cell subsets based on the expression of surface markers.

B cell phenotypes	B220	CD19	CD73	CD80	PD-L2	CD138	IgD
Naïve B cells	+	+					
MBC	+	+	+	+			
MBC	+	+			+		
MBC	+	+	+	+			
MBC	+	+			+		
MBC	+	+	+		+		
MBC	+	+	+	+	+		
MBC	+	+					–
Plasma cells	–	–				+	

Naïve B cells are identified as B220+ and/or CD19+ live lymphocytes; Plasma cells were identified by gating CD138+B220- cells among live cells. Memory B cells (MBC) are determined as CD19+ IgD- live lymphocytes and each Memory B cell subset was identified by gating CD73+, CD80+ and PD-L2+ among IgD-CD19+ live B cells.

### 3.2 Intranasal immunization following E2 treatment induces enhanced secretions of IgG2b and IgG2c in systemic circulation, but not in the vaginal tract

The establishment of MBCs and long-lived plasma cells is key to generating immune responses against WT HSV-2 challenge. A recent study demonstrated that i.n. immunization with TK- HSV-2 elicits a potent B cell response to provide robust protection against WT HSV-2 challenge, though this study did not determine the cellular and histologic source of HSV-2-specific B cells that were primed during immunization (31). One of our goals in this study was to examine the phenotype and local antibody levels in nasal and vaginal mucosa following i.n. immunization to determine the cellular and anatomical source of HSV-2-specific humoral responses. We therefore asked whether E2-treament followed by i.n. immunization could establish B cell and plasma cell populations in the vaginal tract that would ultimately lead to elevated secretions of IgGs in circulation and the vaginal lumen to provide greater protection against HSV-2 challenge.

**Figure 2:**
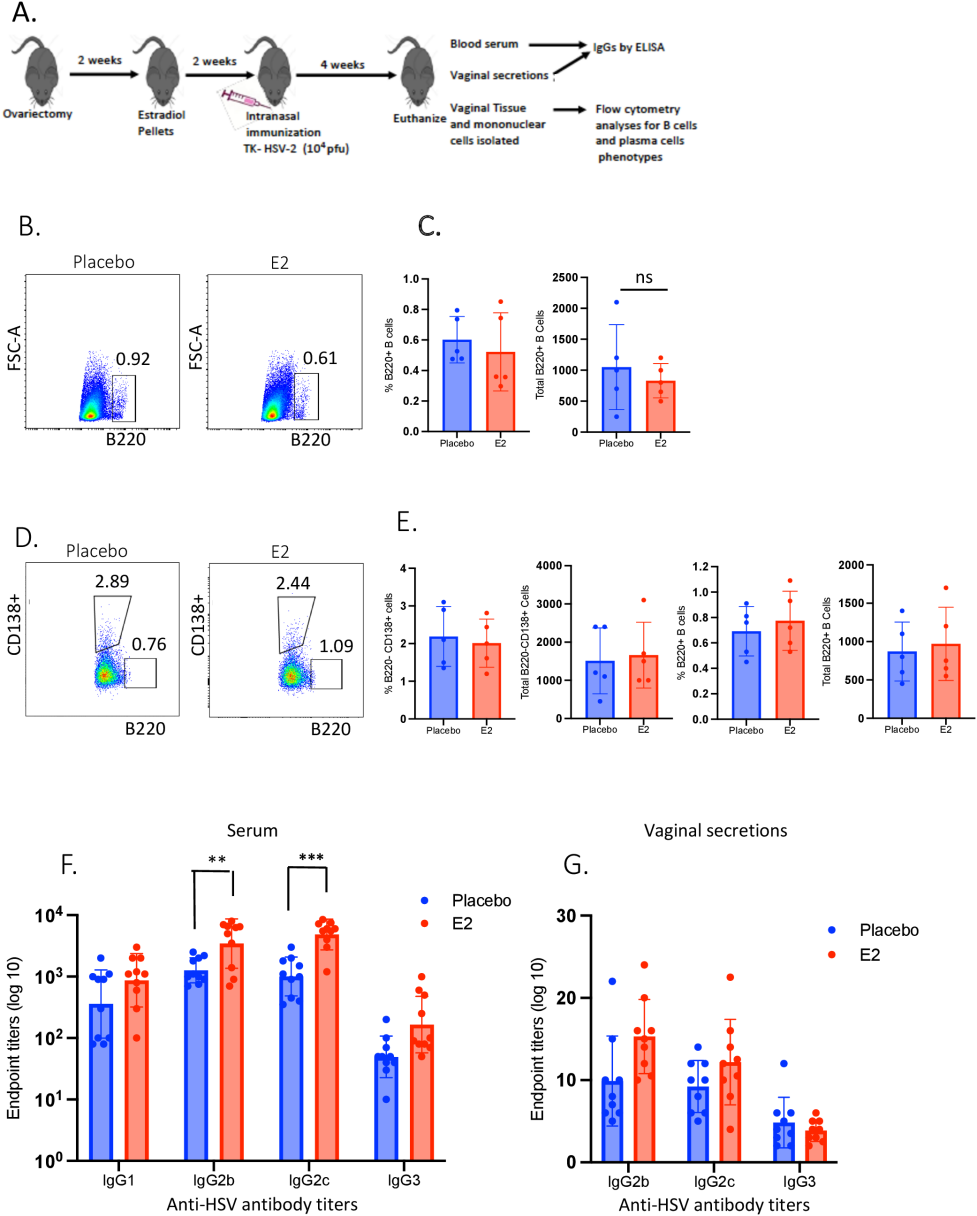
E2-treated mice did not show B cell infiltration in the vaginal mucosa and no increases in HSV-2-specific IgG2b and IgG2c in vaginal secretions, but only in serum, at four weeks post-intranasal immunization. WT OVX mice were implanted with E2 or placebo pellets. Both groups were intranasally immunized with a single dose of TK- HSV- 2 at 10^4^ pfu/mouse. Vaginal washes were collected from day 21–25 post-immunization and pooled for each animal. Serum samples were collected at day 28 post-immunization via orbital bleeding. HSV-2-specific IgG1, IgG2b, IgG2c, IgG3 were measured by ELISA and endpoint titers were determined. Mice were euthanized four weeks post-immunization, vaginal tract tissue collected, and mononuclear cells isolated and analyzed by flow cytometry. **(A)** Experimental lay out; **(B)** Representative dot plots depicting percentages of B220+ B cells gated from total live lymphocytes from the vaginal tract in placebo vs. E2-treated mice; **(C)** Bar graph showing proportion and total number of B220+ B cells; **(D)** Dot plots showing percentages of B220-CD138+ plasma cells and B220+ B cells as gated on total live lymphocytes; **(E)** Bar graphs show proportion and total number of B220-CD138+ plasma cells and B220+ cells; **(F, G)** End point titers of IgG1, IgG2b, IgG2c and IgG3 as measured by ELISA in serum samples **(F)** and vaginal secretions **(G)**. Serum data shown are combined (n=10) from two independent experiments (n=5 mice/group) and vaginal secretions data are pooled (n=9, each sample pooled from two mice) from three independent experiments (n=6 mice/group). Graphs indicate mean ± SEM. Flow cytometry data **(B-E)** shown is a representative of one experiment from three independent experiments showing similar results, the bars indicate mean ± SEM (n=5 mice/group). Data for panels C and E were analyzed using the unpaired, two-tailed *t* test, and panels F and G data were analyzed by two-way ANOVA. **P<0.01; ***P <0.001.

To this end, WT C57BL/6 OVX mice were implanted with E2 or placebo pellets; both groups were intranasally immunized with TK- HSV-2 and animals were sacrificed at four weeks following immunization (**Figure 2A**). Vaginal tracts were collected, and single cells were isolated and analyzed by flow cytometry. Vaginal washes and blood were collected before animals were sacrificed to determine HSV-2-specific IgG subclasses (IgG1, IgG2b, IgG2c and IgG3) by ELISA. Very few B220+ B cells were detected in the vaginal tract of both treatment groups following immunization (**Figure 2B**), and displayed similar frequencies and total number of B220+ B cells (**Figures 2B, C**). Analysis of plasma B cells gated from these B220+ cells revealed only a small percentage of B220-CD138+ plasma cells were present in E2-treated FGT comparable to placebo at four weeks post-immunization (**Figures 2D, E**). Of note, a very low proportion, as well as total number of B220+ cells were determined that were gated from total live lymphocyte (**Figures 2B-E**). Moreover, both E2-treated and placebo control mice displayed comparable frequencies and absolute numbers of B220-CD138+ plasma B cells (**Figures 2D, E**). These findings illustrate that unlike T cells, there is no homing of B cells to the vaginal mucosa following i.n. immunization in the absence of ivag HSV-2 challenge. Importantly, we detected significantly increased levels of HSV-2-specific IgG2c and IgG2b in the serum of E2-treated mice compared to placebo controls (**Figure 2F**). Conversely, very low HSV-2-specific antibodies were detected in the vaginal secretions of E2-treated and placebo mice (**Figure 2G**). These findings indicate that immunization in the nasal mucosa leads to increase in HSV-2-specific antibodies in the serum but not in the vaginal mucosa, even when immunization is in the context of E2.

### 3.3 E2 treatment enhances IgG2c+ plasma cells in the nasal mucosa in intranasally immunized, but not in unimmunized, mice

Next, we evaluated the effect of E2 on the B cell subsets and HSV-2-specific antibody levels locally in the nasal mucosa, following i.n. immunization. We compared functional B cell subsets in the nasal tissue of E2-treated unimmunized naïve mice and E2-treated mice four weeks after i.n. immunization. We hypothesized that E2 treatment would enhance both MBC and plasma cell populations within the secondary lymphoid tissues and effector nasal tissues, respectively.

**Figure 3:**
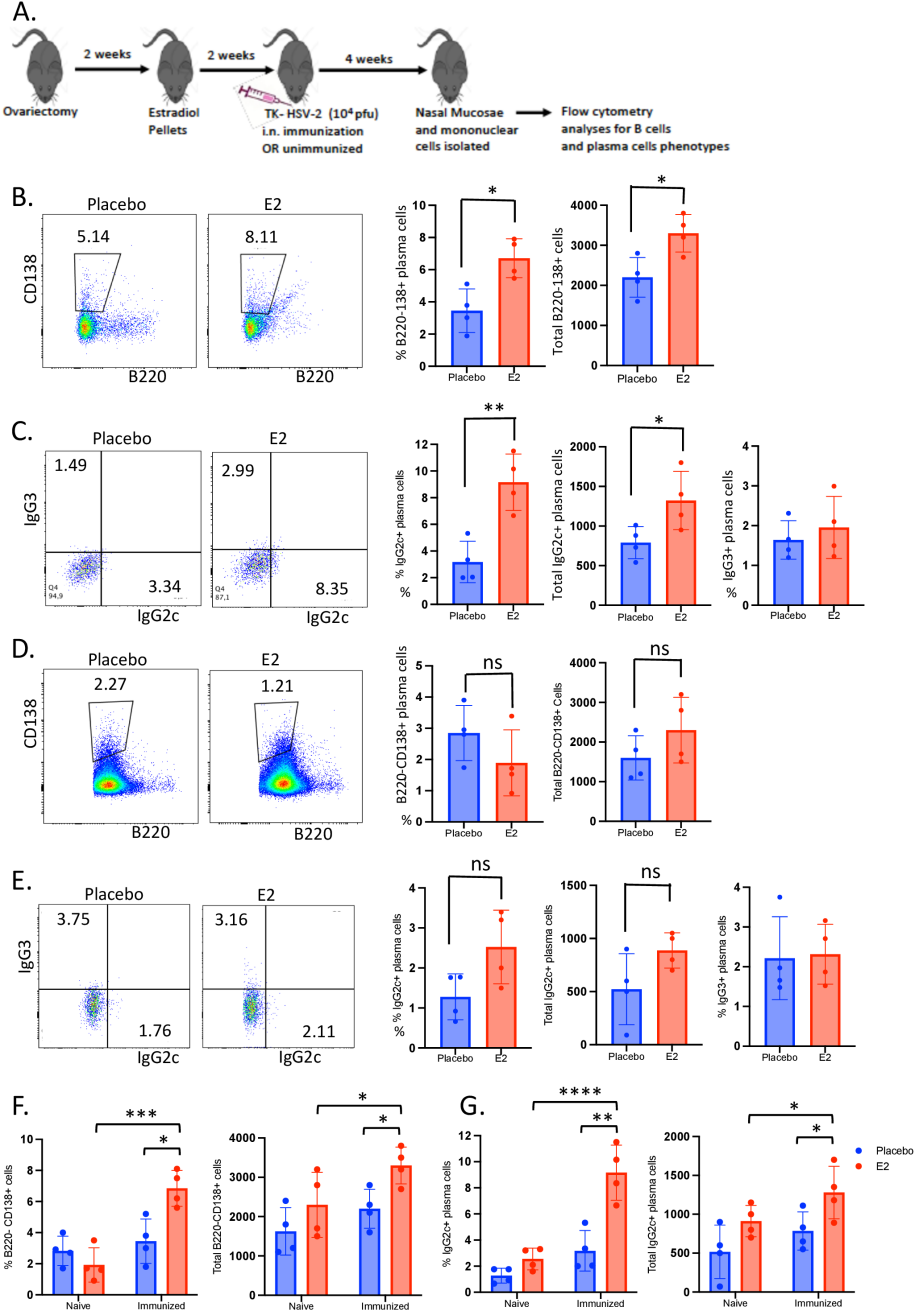
The proportion of IgG2c-secreting plasma cells is enhanced in the nasal mucosa of intranasally immunized, but not in unimmunized naïve, E2-treated mice. WT OVX mice were implanted with E2 or placebo pellets. Mice were either intranasally immunized with a single dose of TK- HSV-2 at 10^4^ pfu/mouse or left unimmunized. Four weeks later, nasal mucosae were collected, pooled, processed and mononuclear cells analyzed by flow cytometry. **(A)** Experimental design. **(B)** B220- CD138+ cells were gated off of total live lymphocytes and their proportion was compared between immunized E2-treated and placebo mice. Bar graphs show the percentages and total numbers of B220-CD138+ plasma cells; **(C)** Representative dot plots show frequency of the IgG2c+ and IgG3+ plasma cell populations, sub-gated from B220-CD138+ plasma cells from panel **(B)**, as compared between immunized E2 treated- and placebo mice. Bar graphs displaying the proportions and total number of B220-CD138+ plasma cells, as well as frequencies of IgG3+ plasma cells. D-E) WT OVX naïve (un-immunized) mice were implanted with E2 or placebo pellets and six weeks later, mice were euthanized, and nasal mucosae was harvested and processed (as described above) for flow cytometry analysis. **(D)** Representative dot plots showing B220- CD138+ plasma cells as gated from total live cells and their proportion was compared between E2-treated and placebo naïve mice. Bar graphs displaying proportion and total number of B220-CD138+ plasma cells. **(E)** Dot plots represent IgG2c+ and IgG3+ plasma cells among B220-CD138+ plasma cells as further gated from B220-CD138+ cells, and the frequency of these populations were compared between E2-treated and placebo naïve mice. Bar graphs show proportion and total numbers of IgG2c+ plasma cells, and frequencies of IgG3+ plasma cells. **(F)** Graphs display the proportions and absolute counts of B220-CD138+ cells as compared between naïve and immunized mice among E2 treated and placebo mice. **(G)** Graphs show proportion and total numbers of IgG2c+ plasma cells as compared between naïve and immunized mice that were previously treated with E2 or placebo pellets. Nasal mucosal tissues were pooled to 4 samples and data shown is from a single experiment as (n=4) representative of the three independent experiments conducted showing similar results (n=8 mice/group). Graphs indicate mean ± SEM. Data for panels **(B-E)** graphs were analyzed by unpaired, two-tailed *t* test, and data for panels **(F, G)** were analyzed by two-way ANOVA.*P <0.05; **P <0.01; ***P<0.001; ****P<0.0001.

To examine this, WT C57BL/6 OVX mice were implanted with either E2 or placebo control pellets and two weeks later, both groups were either left unimmunized or immunized intranasally with TK- HSV-2. Animals were sacrificed four weeks later and nasal mucosae were collected, and mononuclear cells were isolated and analyzed by multicolor flow cytometry for plasma B cell phenotypes. The results indicate that while B220-CD138+ antibody-secreting plasma cells gated from total live lymphocytes were detected within the nasal mucosa both in E2- and placebo-treated mice, significantly increased frequencies and total cell numbers was observed only with E2 treatment compared to placebo controls four weeks post-immunization (**Figure 3B**). Importantly, intracellular staining of lymphocytes revealed that E2-treated immunized mice displayed significantly enhanced frequencies, as well as total number of IgG2c-secreting B220-CD138+ plasma cells compared to placebo control mice (**Figure 3C**). Of note, there was no difference in proportion of IgG3+ plasma cells observed between E2 and placebo groups irrespective of immunized or naïve condition. In contrast, unimmunized naïve mice, irrespective of E2 or placebo treatment, had very low proportions and absolute counts of B220-CD138+ plasma cells (**Figure 3D**), as well as IgG2c-secreting B220-CD138+ plasma cells in the nasal mucosa (**Figure 3E**). Remarkably, analyzing of all four groups of naïve and immunized mice with E2 or placebo treatments together, we observed a significantly enhanced frequencies and total number of B220-CD138+ plasma cells (**Figure 3F**) as well as proportion and total numbers of IgG2c+ plasma cells (**Figure 3G**) in E2-treated immunized mice compared to E2-treated naïve mice (**Figure 3F**). This findings suggest that E2 treatment enhances antibody secreting plasma cells only i.n. immunized mice, but not in unimmunized naive mice despite E2 treatments.

### 3.4 Enhanced viral clearance and plasma B cell antibody responses observed in E2-treated immunized wild-type mice after HSV-2 infection is abrogated in IL-17-/- mice

It has previously been demonstrated that Th17 cells stimulate B-cell proliferation and promote the formation of germinal centers (GCs), leading to isotype switching to IgG1, IgG2a, IgG2b, and IgG3 (33). Further, IL-17 secreted by Th17 cells drives class switch recombination to IgG2a and IgG3, while IL-21 in addition, promotes the switch to IgG2b and IgG1 (33). Notably, E2-induced IFN-γ secreting CD4+ T cell responses following i.n. immunization with TK- HSV-2 has been shown first in the URT within a week and then enriched in the FGT at four weeks post-immunization that were diminished in IL-17-/- mice (12). We therefore examined if the enhanced humoral responses observed in the presence of E2 would be abrogated in the absence of IL-17, which would result in a significant reduction of plasma cells and antibody levels in E2-treated IL-17-/- mice. We addressed this by examining the FGT, peripheral secondary lymphoid tissues and effector sites following i.n. immunization in IL-17-/- mice.

Using the same experimental design as outlined in **Figure 1A**, IL-17-/- OVX mice were implanted with E2 or placebo pellets and two weeks later, both groups were immunized intranasally with TK- HSV-2. Six weeks later, animals were challenged intravaginally with WT HSV-2 and vaginal washes were collected and assessed for viral titers. The NALT, cervical and iliac lymph nodes, nasal mucosa, spleen, and vaginal tract were collected, and single cells were isolated and analyzed by flow cytometry. Vaginal washes and blood were collected before animals were sacrificed to determine HSV-2-specific IgM, IgG1, IgG2b, IgG2c and IgG3 by ELISA.

**Figure 4:**
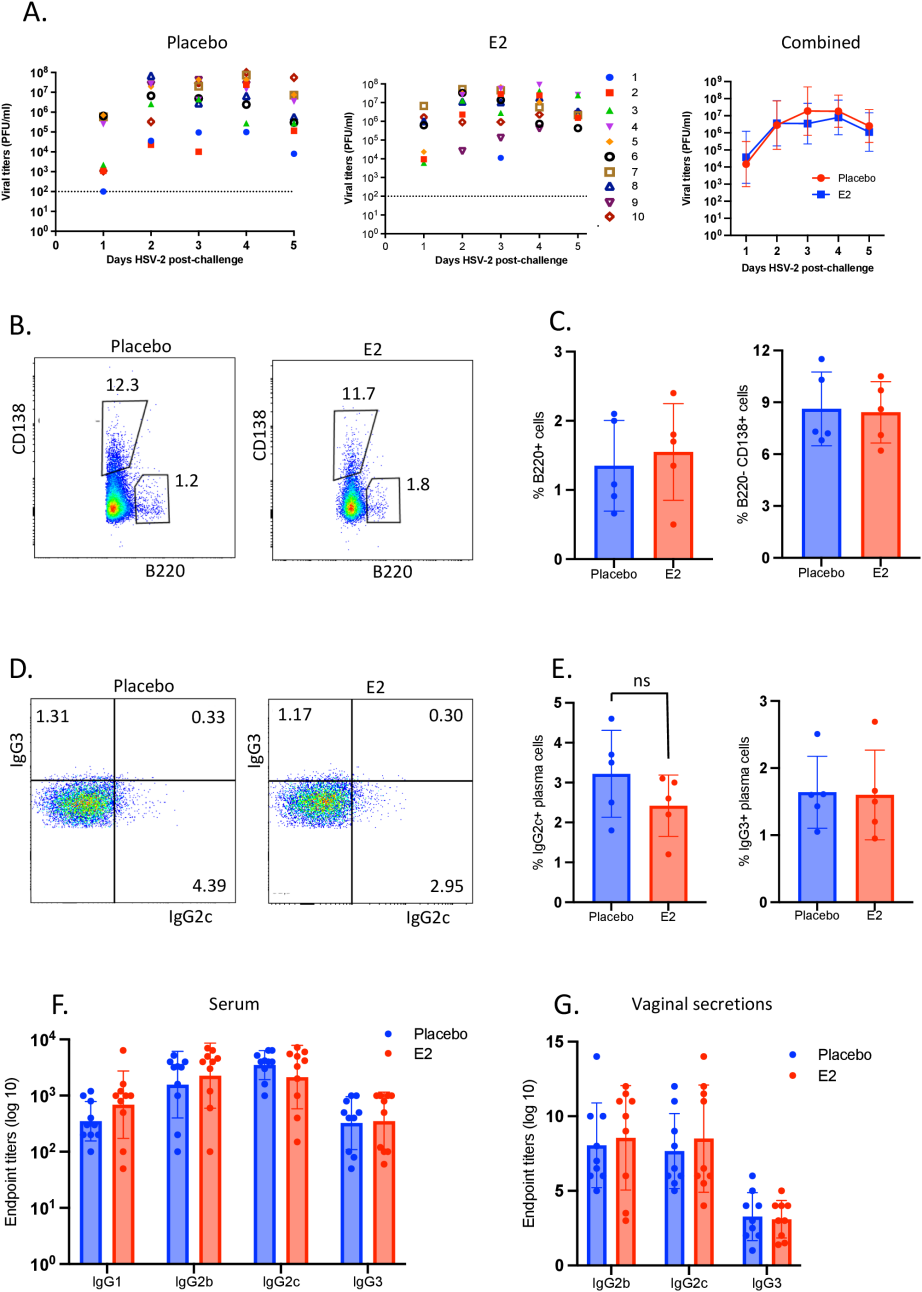
E2 treatment had no effect on virus clearance, B220-CD138+ plasma cells and serum or vaginal IgG2b and IgG2c levels in immunized IL-17-/- mice challenged with HSV-2. IL-17-/- OVX mice were implanted with E2 or placebo pellets and after two weeks, both groups were intranasally immunized with TK- HSV-2 at 10^4^ pfu/mouse. Six weeks later, both groups were challenged intravaginally with WT HSV-2 at 10^4^ pfu/mouse. Vaginal washes were collected daily up to 5 days post-challenge for viral shedding. Vaginal tissues were collected at day 5 post-challenge, and mononuclear cells were isolated and analyzed by flow cytometry. Serum samples and vaginal washes were collected at day 5 post-challenge to assess HSV-2-specific IgG1, IgG2b, IgG2c, IgG3 by ELISA and endpoint titers were determined. **(A)** Vaginal washes collected daily for 5 days post-challenge were assessed for viral titers and results are depicted in separate panels for E2 and placebo groups, with the right-most graph showing the combined viral titers to compare between E2 and placebo treatments. Data for panels B to E, IL-17-/- mice were treated similarly as shown for experimental design in **Figure 3A** and nasal mucosal cells were analyzed. **(B)** Representative dot plots showing percentages of B220+ B cells and B220- CD138+ plasma cells as gated from total live lymphocytes; **(C)** Proportion of B220+ B cells and B220-CD138+ plasma cells were compared between E2-treated and placebo mice; **(D)** Representative dot plots showing the percentages of IgG2c+ and IgG3+ plasma cells as gated on B220-CD138+ plasma cells from E2-treated and placebo mice; **(E)** Percentages of IgG2c+ and IgG3+ plasma cells as compared between E2-treated and placebo mice. **(F, G)** HSV-2-specific IgG antibody titers at 5 days post-challenge in **(F)** serum samples and **(G)** vaginal secretions. HSV-2-specific IgG1, IgG2b, IgG2c, IgG3 were measured by ELISA and endpoint titers were determined. Viral titers **(A)** and serum samples **(F)** shown are combined (n=10) from two independent experiments (n=5 mice/group), while vaginal secretions are pooled (n=9, each sample was pooled from 2 mice) from three independent experiments (n=6 mice/group). Graphs indicate mean ± SEM. Flow cytometry data shown is from a single experiment as representative of the three independent experiments with similar results. Data for panel **(A, C, D)** were analyzed by the unpaired, two-tailed *t* test, and data for panels F and G were analyzed by two-way ANOVA.

We first assessed the viral shedding in E2 treatment as compared to placebo controls. E2-treated IL-17 -/- mice that were immunized intranasally with TK- HSV-2 and subsequently challenged intravaginally with WT HSV-2 6-weeks later displayed vaginal viral shedding similar to placebo control mice (**Figure 4A**). We next examined the antibody-secreting plasma cells residing the nasal mucosa of E2-treated and placebo IL-17-/- mice at four weeks post-immunization. Both E2 and placebo groups showed comparable proportions of B220-CD138+ plasma cells in the nasal mucosa (**Figures 4B, C**). Of note, similar to WT mice, only fewer B220+ cells, as well B220-CD138+ plasma cells as gated from total live lymphocytes were present in the FGT of both E2-treated and placebo IL-17-/- mice (**Supplementary Figure S3**). Furthermore, intracellular staining for IgG2c and IgG3 revealed that the proportion of both IgG2c and IgG3 expressing B220-CD138+ plasma cells was not significantly different between E2-treated and placebo groups (**Figures 4D, E**), which further reiterates the requirement of IL-17 and augmented Th17 cells in E2-enhanced B cell immunity.

Next, the levels of HSV-2-specific IgG1, IgG2b, IgG2c, and IgG3 at six weeks post-immunization in the serum and vaginal washes after HSV-2 challenge were measured and compared between E2-treated and placebo IL-17-/- mice. In contrast to the findings observed with WT mice, no difference was observed between E2-treated and placebo IL-17-/- OVX mice with respect to serum and vaginal HSV-2-specific IgG2b and IgG2c levels (**Figures 4F, G**).

### 3.5 E2 treatment markedly enhances the proportion of memory B cells in the spleen and iliac lymph nodes four weeks after TK- HSV-2 immunization

Our previous experiments indicated that the cellular source of HSV-2-specific antibodies detected post-immunization and challenge lies outside of the FGT. We therefore wanted to examine other immunological sites to characterize MBCs and antibody-secreting plasma cells induced following i.n. immunization. A heterogenous population of MBCs are induced upon antigenic stimulation with differential effector functions. In mice, multiple strategies have been employed to identify surface markers associated with MBCs and MBCs can be divided into subsets based on the expression of CD73, CD80, and PD-L2 (41–43) (**Table 1**). These three markers were used in our experiments to identify MBC subsets. Since MBCs are defined by the heterogenous expression of CD73, CD80, and PD-L2, we therefore measured the frequency and total number of individual subsets (individual frequency and total number, respectively) as well as combined frequencies of all subsets which lie within CD19+IgD- memory B cell parent populations. We further evaluated whether these populations are altered by E2 treatment and ultimately lead to the superior anti-HSV-2 responses observed.

**Figure 5:**
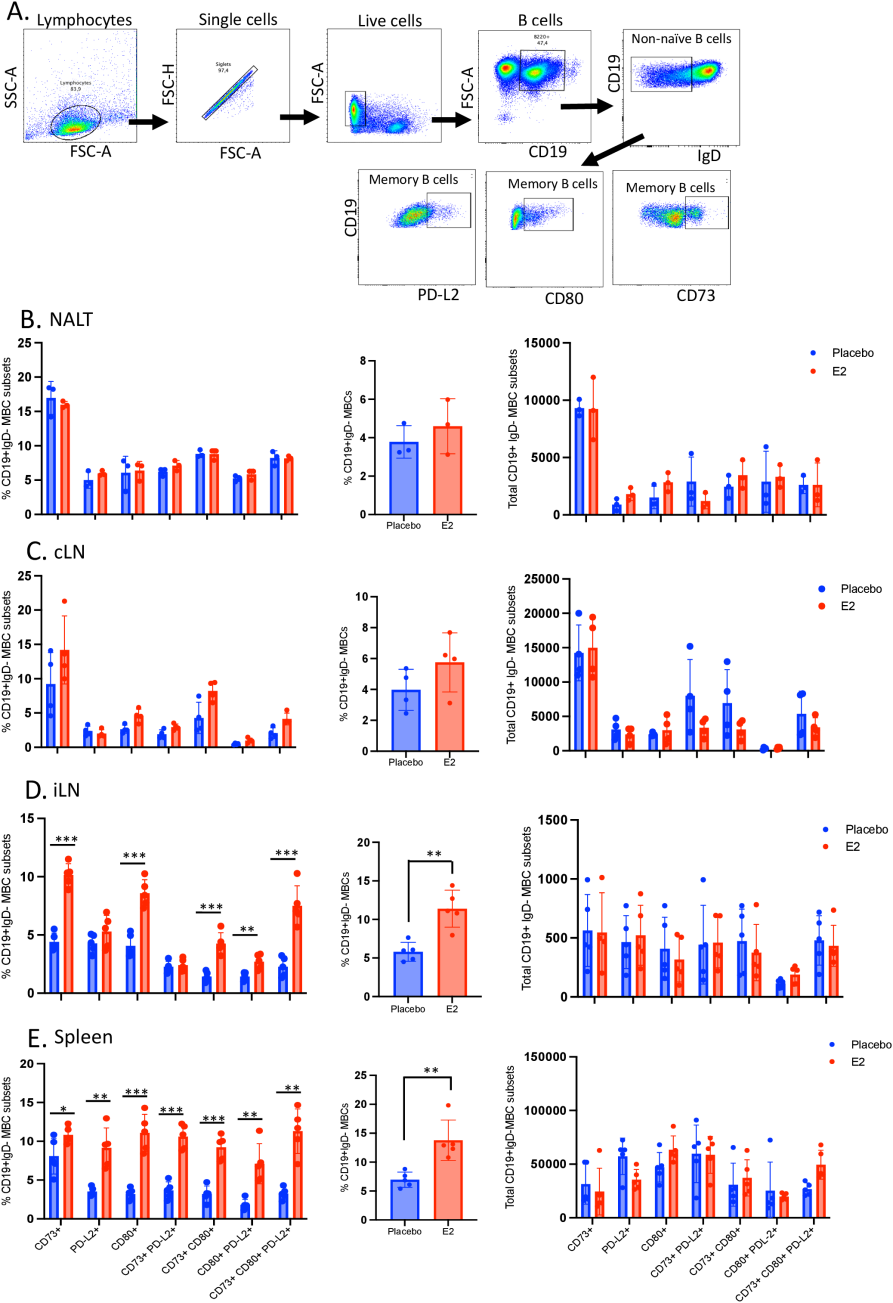
E2 treatment enhances memory B cell phenotypes within peripheral secondary lymphoid tissues following intranasal immunization in C57BL/6 OVX mice. WT C57BL/6 OVX mice were implanted with E2 or placebo pellets. Both groups were intranasally immunized with TK- HSV-2 at 10^4^ pfu/mouse. Four weeks later, nasal associated lymphoid tissue (NALT), cervical lymph nodes (cLN), iliac lymph nodes (iLN) and spleen were collected, pooled, processed, stained with a panel of antibodies, and analyzed by flow cytometry. **(A)** Memory B cells were gated from total live lymphocytes as CD19+IgD- cells and identified by the heterogenous expression of CD73, CD80, or PD-L2. The differences in percentages of individual subsets (left panels), the combined frequency counted as CD19+IgD- populations for all subsets (middle panels), and total number of memory B cells (right panels) following intranasal immunization was examined as displayed in **(B)** NALT, **(C)** cervical lymph nodes, **(D)** iliac lymph nodes, and **(E)** spleen. Middle panel shows % CD19+ IgD- MBCs gated on live cells as the parent population of the subsets. Data shown is from one representative experiment out of three independent experiments showing similar results. NALT tissues were pooled to 3 samples (n=3) and cLN samples were pooled to 4 samples (n=4) from n=6 mice to get enough cells for flow cytometry analyses, and for iLN (n=5) and spleen (n=5). Graphs indicate mean ± SEM. Data for all middle panels were analyzed by the unpaired, two-tailed *t* test, and data for all left and right panels **(A-D)** were analyzed by two-way ANOVA. *P<0.05; **P <0.01; ***P <0.001.

At four weeks post-immunization, MBCs were found within the NALT, cervical and iliac lymph nodes and spleen, both in E2-treated and placebo mice. In a parallel experiment, we found diminished formation of MBCs in the NALT and overall lower levels in lymphoid tissues in immunologically naïve mice (**Supplementary Figure S4**). The NALT and cervical lymph nodes isolated from E2-treated mice had comparable levels of individual subset frequency, combined frequency and total number of MBCs to those isolated from control mice (**Figures 5B, C**). However, the combined frequency of MBCs was significantly greater in the iliac lymph nodes and spleen isolated from E2-treated mice (**Figures 5D, E**). When analyzed as individual subsets, similar significant increases in frequency were likewise observed for most MBC populations (**Figures 5D, E**). The only subsets not in line with this observation were PD-L2+ and CD73+PD-L2+ MBCs isolated from iliac lymph nodes. Of note, the significant differences observed in the iliac lymph nodes and spleen were mainly the frequency but not total number of cells seen in MBC subsets, indicative of enrichment of these populations (**Figures 5D, E**).

In a separate sets of experiments, we investigated whether E2 treated immunized mice following HSV-2 challenge also show enhanced memory B cell profiles. E2 treated or placebo mice after 4 weeks of immunization were challenged ivag with HSV-2 and cells from peripheral lymphoid tissues were analyzed by flow cytometry for memory B cells phenotypes. Notably, pronounced enhancement of most memory B cell subsets frequencies were determined in iliac lymph nodes (**Supplementary Figure S5A**, left panel) and only CD80+PD-L2+ subset found in spleen (**Supplementary Figure S5B**, left panel) in E2 treated immunized, but not in placebo mice after HSV-2 challenge. Of note, there were no enhancements in the formation of memory B cells in in NALT or cLN irrespective of E2 or placebo treatments (data not shown). However, we determined no differences in cell numbers between E2 vs placebo treatments (**Supplementary Figures S5A, B**, right panels).

We further examined if intravaginal HSV-2 infection in E2 treated or placebo immunized mice leads to the development of HSV-2 specific memory B cells in the vaginal mucosa. For this, E2 or placebo treated immunized mice were challenged with HSV-2 and vaginal tract mucosal cells were isolate and analyzed by flow cytometry for HSV-2 specific memory B cells. More specifically we evaluated CD19+IgD- memory B cells that were positive for HSV-specific IgG. Importantly, we observed a significantly enhanced proportions of IgG+CD19+IgD- memory B cells in the vaginal mucosa in E2 treated immunized mice compared to E2-treated naïve mice (**Supplementary Figure S5C**, left panel). Similarly, E2 treated immunized mice after HSV-2 infection showed significant enrichment of IgG+CD19+IgD- memory B cell populations in vaginal tract compared to naïve counterparts (**Supplementary Figure S5C**, right panel). We also determined a greater proportion of IgG+CD19+IgD- memory B cells in E2 treated immunized mice compared to immunized placebo mice (**Supplementary Figure S5C**). These findings provide additional evidence that E2 treatment together with i.n. immunization for 4 weeks followed by HSV-2 challenge establishes HSV-2 specific memory B cells in the FGT that could plausibly precede to HSV-2 specific antibody production in the vaginal lumen.

### 3.6 E2-induced memory B cell phenotypes observed following i.n. immunization in WT mice are abrogated in IL-17-/- mice

In line with the findings that E2 enhances IFN-γ- and IL-17-secreting CD4+ T cells within the URT and FGT at four weeks post-immunization in WT but not found in IL-17-/- mice (12), here we wanted to see if the formation of MBCs observed in E2 treated immunized WT mice also requires IL-17. We addressed this by examining the local inductive sites in the URT, peripheral secondary lymphoid tissues and effector sites following i.n. immunization of IL-17-/- mice. Using the same experimental design as outlined in **Figure 2A**, IL-17-/- mice were implanted with E2 or placebo pellets and two weeks later, both groups were immunized intranasally with TK- HSV-2. Animals were sacrificed four weeks post-immunization, and the NALT, cervical and iliac lymph nodes and spleen were collected and mononuclear cells were analyzed by multicolor flow cytometry for MBC phenotypes.

**Figure 6:**
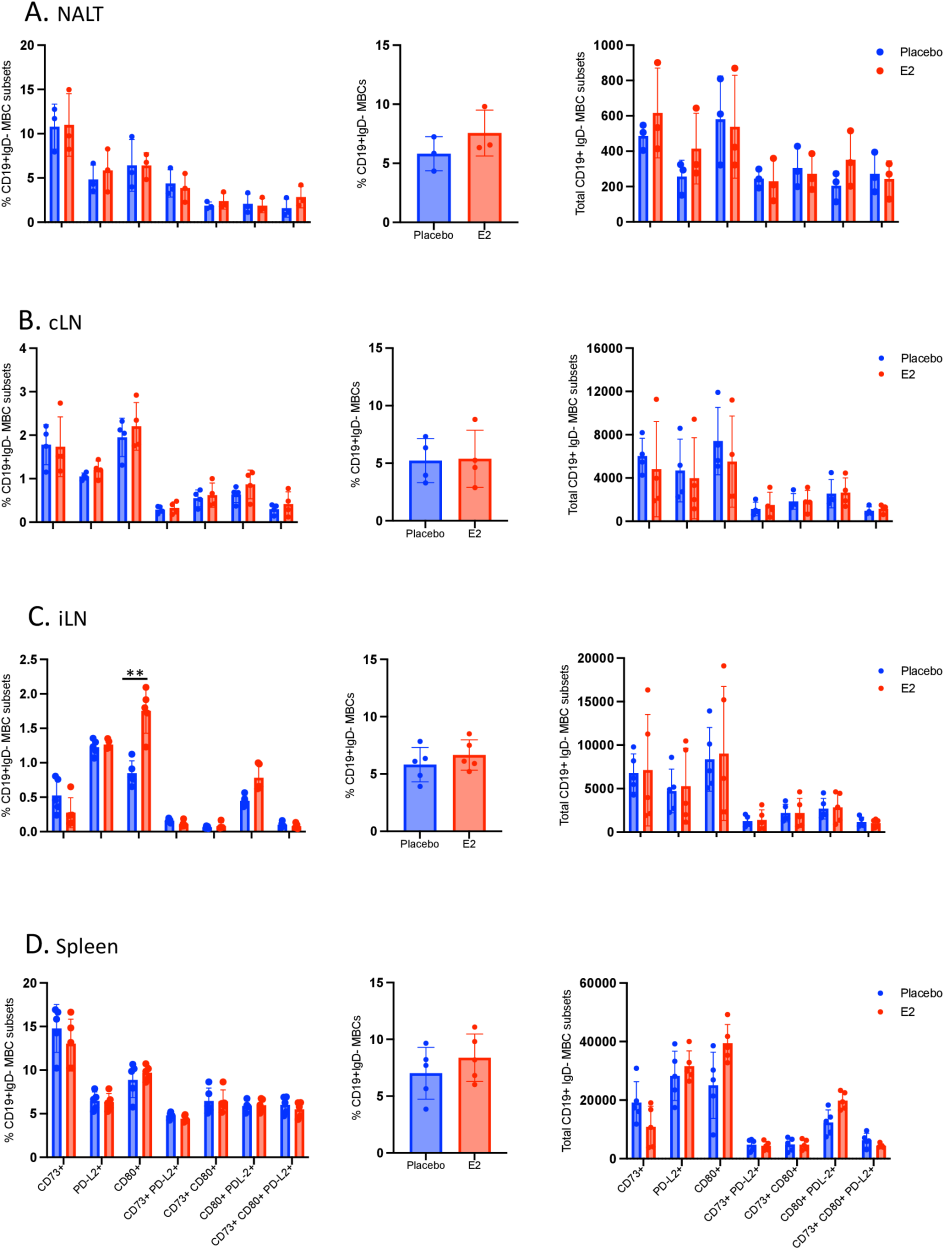
The effects of E2 on enhanced memory B cell formation in secondary lymphoid tissues following intranasal immunization is abrogated in IL-17-/- mice. Ovariectomized IL-17-/- mice were implanted with E2 or placebo pellets. Both groups were immunized intranasally with TK- HSV-2 at 10^4^ pfu/mouse. Four weeks later, NALT, cervical lymph nodes, iliac lymph nodes and spleen were collected, pooled, processed, stained with a panel of antibodies, and examined by flow cytometry. Memory B cells were gated from total live lymphocytes as CD19+IgD- cells and identified by the heterogenous expression of CD73, CD80, or PD-L2 as shown in **Figure 5A**. The differences in percentages of individual subsets (left panels), the combined percentages of CD19+IgD- populations that include all subsets (middle panels), and the total number of memory B cells (right panels) following intranasal immunization are shown in **(A)** NALT, **(B)** cervical lymph nodes, **(C)** iliac lymph nodes, and **(D)** spleen. Middle panel shows % CD19+ IgD- MBCs gated on live cells as the parent population of the subsets. Data shown is from one representative experiment out of three independent experiments showing similar results. NALT tissues were pooled to 3 samples (n=3) and cLN samples were also pooled to 4 samples (n=4) from n=6 mice/group to get enough cells for flow cytometry analyses. iLN (n=5) and spleen (n=5). Graphs indicate mean ± SEM. Data for middle panels were analyzed by the unpaired, two-tailed *t* test, while data for all left and right panels **(A-D)** were analyzed by two-way ANOVA. **P <0.01.

We observed similar profiles of memory B cell subsets within the NALT and cLN in IL-17-/- mice displaying no noticeable differences between E2-treated and placebo immunized mice irrespective of individual or combined frequencies and absolute counts of MBCs (**Figures 6A, B**), similar trend but reduced populations as determined in WT mice. Importantly, unlike the significant enhancement of MBC markers observed in the iLN and spleen in E2-treated immunized WT mice versus placebo mice, IL-17-/- mice displayed no difference but a similar MBC profile to their placebo counterpart (**Figures 6C, D**). Specifically, the NALT, cervical and iliac lymph nodes and spleen isolated from E2-treated IL-17-/- mice had comparable individual frequency, combined frequency and total number of MBCs (**Figures 6A–D**). Notably, regardless of treatment group examined, IL-17-/- mice had very little to no double or triple positive MBC subsets (CD73+CD80+, CD73+PD-L2+, CD80+PD-L2+, or CD73+CD80+PD-L2+) in the NALT, cLN, iLN and spleen (**Figures 6A–C**). These results indicate that the differentiation of B cells into MBC subsets is impaired in the absence of IL-17, meaning that E2-induced enrichment of MBCs occurs in an IL-17 dependent pathway.

## 4 Discussion

Our present study provides novel evidence that E2 treatment elicits robust reduction in viral titers which is inversely associated with the increased levels of HSV-2 specific antibodies in the serum and vaginal tract in wild-type OVX mice challenged with WT HSV-2 that were previously immunized i.n. with TK- HSV-2 for 6 weeks. We further demonstrate that E2 treatment significantly enhanced the frequencies of B220-CD138+ plasma B cells in the vaginal mucosa that likely induce HSV-2-specific IgG2b and IgG2c antibodies in the vaginal lumen and serum. Importantly, E2 treatment appeared to increase the subsets of CD19+ IgD- memory B cells with heterogenous expression of CD73, CD80, and PD-L2 within the iliac lymph nodes and spleen four weeks post-immunization. Remarkably, we elucidate that the E2-mediated enhanced viral clearance, increased memory B cells and HSV-2 specific antibody responses are abrogated in in IL-17-/- mice indicating requirement of IL-17-mediated pathway.

Earlier studies aimed at developing HSV-2 vaccine utilizing animal models have focused on cell mediated-immunity for generating immunological memory against the virus following vaccination. However, optimal vaccine-induced protection requires antibody-mediated immunity to prevent productive viral infection and establishment of latency (14, 31). Antibody-based vaccines against HSV-2 have failed to confer protection in humans (44) since none of the vaccines induced enough antibodies that were required within the vaginal lumen to protect from HSV-2 infection (45). In addition, the routes of mucosal immunizations determine the level of protection, while ivag immunization confers complete protection by establishing high levels of CD4 T_RM_ cells in the FGT (30), intranasal immunization confers protection by inducing both T cells and HSV-2-specific antibodies which neutralize the virus in the dorsal root ganglia (30). Although ivag immunization proves superior in eliciting robust local immune responses in the FGT, however, clinical acceptability has not been evaluated (46). On the other hand, intranasal immunization has been proven as an effective strategy in human clinical settings (46). Importantly, how and where the antibody responses are elicited by i.n. immunization remains unclear. Therefore, in this study, we sought to understand B cell responses following i.n. immunization and identify strategies to maximize B cell responses in the FGT. We evaluated the efficacy of exogenous E2 as a novel strategy to accelerate antiviral humoral immunity in the FGT.

In this study we observed a significantly decreased vaginal viral shedding in E2 treated, but not placebo, i.n. immunized mice challenged with HSV-2 that is inversely associated with levels of IgG2b and IgG2c antibodies both in the vaginal lumen and serum. Currently, it is not clear whether the humoral protection conferred following i.n. immunization originates from the antibody-secreting long-lived plasma cells primed during immunization or MBCs that infiltrate the vaginal mucosa post-immunization and differentiate into plasma cells upon ivag viral challenge. E2 treatment induced greater proportions of B220-CD138+ plasma cells in the FGT compared to placebo controls (**Figure 1F**) and these B220-CD138+ plasma cells within the FGT lead to enhanced production of HSV-2-specific IgG2b and IgG2c antibodies in the vaginal lumen (**Figures 1G, H**) that resulted reduction in viral titers compared to placebo mice. These findings indicate that E2 treatment induces a microenvironment within the FGT which preferentially enhances the recruitment of antibody-secreting plasma cells rather than MBCs or naive B cells upon ivag HSV-2 challenge.

Although we did not explore the mechanisms by which B cells were recruited into the FGT upon ivag challenge, it is reasonable to speculate that the mechanism may likely be similar to that demonstrated by Oh et al. (17). In that study, they demonstrated that CD4+ TRM cells established within the FGT post-ivag immunization secrete IFN-γ upon ivag challenge and induce the expression of CXCR3 ligands (CXCR9 and CXCR10) within the vaginal mucosa, resulting in migration of B cells into the vaginal lumen, which secrete virus specific-IgGs through a CXCR3-dependent pathway. Although the route of immunization was different between our (i.n.) and Oh et al. (17) (ivag) studies, recruitment of B cells from the effector sites to the vaginal lumen follows a similar strategy, which is through a CD4 T_RM_ IFN-γ CXCR3 dependent manner, irrespective of route of immunization. In support of this, alveolar macrophages are known as the key initiator for humoral recall response through the secretion of IFN-γ and CXCR3 ligands, which activate the recruitment of CXCR3+ resident memory B cells into the foci of infected cells (48).

In line with our previous observation that E2 treated immunized mice showed increased Th17 and Th1 T_RM_ cells in the FGT led robust protection against HSV-2 challenge (12), we therefore hypothesized that E2 treatment in immunized mice would enrich plasma B cells in the FGT leading to secretions of HSV-2 specific IgG2b and IgG2c antibodies in the vaginal lumen. We first characterized plasma B cell and HSV-2-specific antibody responses at four weeks post-i.n. immunization between E2-treated and placebo mice. Notably, no infiltration of plasma or B cells found in the FGT after 4 weeks of immunization (**Figures 2B–E**). This could be the underlying reason that a traces to no HSV-2-specific IgG2b or IgG2c antibodies detected in the vaginal lumen (**Figures 2F, G**). These findings indicate that B cell responses elicited by i.n. immunization primarily originate outside of the FGT. This poses a number of clinical implications. First, our observations complement the findings of Oh et al. (17) and reveal that regardless of the route of mucosal immunization, B cells are only recruited into FGT upon local secondary exposure. Moreover, high levels of HSV-2-specific antibodies within circulation following immunization do not translate to their presence within the vaginal mucosa. These observations help explain, at least in part, the underlying reason why antibody-mediated HSV-2 vaccines developed to date have all failed to confer protection in humans (47). The absence of these antibodies in the local tissue upon viral entry into the FGT renders the vaccines ineffective (48, 49). Results from our study show that i.n. immunization is equally effective as ivag immunization with respect to generating antibody-mediated responses that has been previously demonstrated by Parr and Parr (50) and Oh et al. (17). Parr and Parr reported that both i.n and ivag immunization had comparable levels of recruitment of lymphocytes including B cells in the vaginal tract, while Oh et al. (17) in a recent study also detected a significantly elevated levels of HSV-2 specific IgG2b and IgG2c antibodies in the vaginal lumen in mice challenged with HSV-2 that were previously ivag immunized for five weeks. Therefore, given better utility in a clinical setting, future studies should consider the i.n. route of immunization for eliciting effector B cell subsets within the FGT. One such strategy that may be successful in accomplishing this goal is “the prime and pull” strategy described by Shin and Iwasaki (51). More specifically, CXCR3 ligands may be applied to the vaginal cavity, a few weeks after i.n. immunization, to “pull” the effector B subsets into the FGT and generate luminal HSV-2-specific antibodies.

A recent study demonstrated that upon i.n. immunization, IgG responses were initially detectable in the NALT and cervical lymph nodes and only appeared in the spleen at a later timepoint (52). This is very likely the case in our study, where i.n. immunization in the presence of E2 leads to an increased proportion of CD4+ TRM cells within local inductive sites which may result in an enhanced rate of B cell differentiation and increased proportion of effector B subsets, which then migrate to other sites. In support of this mechanism, we also found that E2 treatment significantly enhanced the establishment of IgG2c+CD138+B220- plasma cells within the nasal mucosa (mucosal effector site) (**Figure 3C**). Additionally, this finding substantiated our earlier observations and revealed that the enhanced HSV-2-specific IgG2c detected in the serum of E2-treated mice likely stems from the augmented IgG2c-secreting plasma cells located within the effector sites of the URT. However, additional studies required to confirm this likely link between the E2-mediated enrichment of plasma B cells in the URT and elevated HSV-2 specific IgG2c in serum upon i.n. immunization. Finally, to ensure that the E2-mediated enhancements were indeed specific to i.n. immunization and did not occur in a non-specific manner, we conducted a parallel experiment in which neither groups were immunized intranasally. As expected, none of the E2-mediated enhancements that were seen post-i.n. immunization were observed in immunologically naive mice. Altogether, these results indicate that functional B cell responses are induced with E2 treatment only upon i.n. immunization. Additionally, this finding complements our earlier observations that the enhanced HSV-2-specific IgG2c detected in the serum of E2-treated mice stems from the augmented IgG2c-secreting plasma cells localized within the effector sites of the URT.

The memory compartment of the humoral immune system is composed of long-lived plasma cells and memory B cells. The functional differentiation of these two compartments would allow us to define the roles of MBCs in protection from reinfection with a specific pathogen (53). In order to define cellular or tissue sources of effector memory B cells subsets induced by E2 treatment following immunization, we analyzed cells from URT (NALT), cLN, iLN and spleen. Importantly, E2 treated immunized mice exhibited greater proportion of memory B cell subsets in the spleen and iliac lymph nodes, which were not induced in immunologically naïve mice. This indicates preferential compartmentalization of MBCs in different tissues. While MBCs mainly compartmentalized within the local and peripheral secondary lymphoid tissues (spleen and iliac lymph nodes), the antibody-secreting plasma cells on the other hand primarily resided within the nasal mucosa. These findings indicate that these plasma cells are likely cellular source of HSV-2-specific antibodies detected in the serum at four weeks post-i.n. immunization. In line with findings from previous studies, these antibodies were predominately of IgG2b and IgG2c subclasses (30, 54). Outside of the FGT, we observed E2 treatment enhanced both MBC and plasma cell subsets within distinct sites four weeks post-i.n. immunization, since iliac lymph nodes and spleens isolated from E2-treated mice had significantly greater proportions of individual MBC subsets (**Figures 5D, E**). We further determined that E2 treated mice after HSV-2 challenge that were i.n. immunized 4 weeks previously showed significantly greater proportion of memory B cell subsets mostly in iLN and only CD80+PD-L2+ subset in spleen tissue (**Supplementary Figures S5A, B**, left panels). Remarkably, E2-treated immunized (4 weeks) but not naïve mice after HSV-2 challenge established increased HSV-2 specific IgG+CD19+IgD- memory B cells in the vaginal tract (**Supplementary Figure S5C**). Of note, while the same MBC subsets were present within the local inductive sites (NALT and cervical lymph nodes), the E2-mediated enhancement was not observed (**Figures 5B, C**). As mentioned earlier, our lab recently discovered that i.n. immunization in the presence of E2 leads to greater establishment of IFN-γ+ and IL-17+ CD4+ TRM cells only within the NALT and cervical lymph nodes and not the iliac lymph nodes or spleen (12). These E2-mediated enhancements were observed as early as one week post-i.n. immunization and persisted until at least for four weeks. We believe that a small subset of these CD4+ TRM cells, which reside within these sites permanently, may serve to help B cells differentiate to functional effector subsets. Upon differentiating into MBCs or plasma cells, these effector B cells may then migrate from the NALT and cervical lymph nodes to peripheral secondary lymphoid tissues and effector mucosal sites (52, 55).

In fact, enrichment of these differentiated effector memory B cells could be antibody-secreting plasma cells in the FGT since they exhibit more memory-like phenotype (co-expressing CD73, CD80, and PD-L2) and can differentiate into plasma cells upon infiltrating the FGT (56). Regardless, the findings from the current study illustrate that by day 5 post-ivag challenge, plasma cells constitute the only B cell population within the FGT in E2-treated mice which readily secrete luminal HSV-2-specific antibodies and contribute to early protection from the virus. Furthermore, antibodies secreted by these cells are thought to be of higher affinity for the virus. This is thought to occur due to the antigen affinity dependent manner by which MBC and plasma cell differentiation occurs. As described in detail by Zuccarino-Catania et al. (42), of the B cells that enter the GC, B cells with low affinity B cell receptors (BCR) receive little to no cognate T cell help and are driven toward a MBC phenotype with less memory-like phenotype (do not co-express CD73, CD80, and PD-L2). Conversely, those with high affinity BCR strongly interact with cognate T cells and differentiate into more memory-like MBCs and plasma cells (42, 57). Overall, these findings provide further insight into the immunological mechanisms by which E2 treatment enhances protection against HSV-2. In this study, we discovered how the augmentation of vaccine-induced immunity in the presence of E2 translates to the establishment of antibody secreting plasma cells within the FGT following ivag challenge, which leads to enhanced levels of luminal HSV-2-specific IgG2b and IgG2c and ultimately, confers superior protection against HSV-2.

Our previous studies revealed that the superior protection observed with E2 treatment was related to increased Th1 and Th17 immunity in the FGT (10). Specifically, we found this to be dependent on the secretion of IL-1β by vaginal CD11c+ DCs which augmented Th17 responses within the FGT (10). Subsequently, the enhanced Th17 response augmented IFN-γ+ CD4+ T cell immunity against HSV-2, which is critical for protection (11, 58). In the current study, E2 treatment appears to enhance HSV-2-specific IgG2b and IgG2c levels. In order for a B cell to undergo recombinant isotype switching to IgG, the help of its cognate T cell is required (59). In this study, we wanted to examine if the absence of IL-17 would result in abrogation of E2-mediated enhancement of the B cell responses observed after i.n. immunization and ivag challenge. We found that the E2-mediated enhanced protection observed in WT mice was abolished in the absence of IL-17 (**Figure 4A**) and this correlated with decreased antibody-secreting plasma cells, and IgG2b and IgG2c antibodies in the FGT of both E2-treated and placebo mice (**Figure 4**). Moreover, E2-treated and placebo IL-17-/- mice displayed comparable proportion of MBCs across all of their secondary lymphoid tissues and IgG2c+-secreting plasma cells within the nasal mucosa. Therefore, these findings indicate that E2-mediated enhancement of B cell responses were impaired in the absence of IL-17, likely due to diminished IFN-γ+ CD4+ T cells mediated in the absence of Th17 cells.

In summary, we conclude that i.n. immunization with TK- HSV-2 in the presence of E2 leads to enrichment of heterogenous CD19+IgD- MBC subsets within secondary lymphoid tissues and antibody-secreting plasma cells within the nasal effector sites. Subsequently, upon ivag exposure to WT HSV-2, these enhanced responses bolster a more rapid and strong anti-viral protective response within the FGT by establishing increased levels of HSV-2-specific IgG within the vaginal lumen and ultimately confer superior protection. Our current results revealed a potential strategy to augment robust antibody secretions into the FGT through E2 treatment in an IL-17-dependent pathway. This understanding may be leveraged to develop effective antibody-mediated vaccine against STIs, including HSV-2.

## Data Availability

The original contributions presented in the study are included in the article/**Supplementary Material**. Further inquiries can be directed to the corresponding author.

## Supplementary material

Supplementary Table 1 Characteristics of B cell subsets based on the expressions of surface markers. Naïve B cells are identified as B220+ and/or CD19+ live lymphocytes; Plasma cells were identified by gating B220-CD138+ cells among live cells. Memory B cells (MBC) are determined as CD19+ IgD- live lymphocytes and each Memory B cell subset was identified by gating CD73+, CD80+ and PD-L2+ among CD19+IgD- live B cells.

Supplementary Figure 1 Effects of E2 treatment on immunized mice challenged with HSV-2 on IgGs production in serum and vaginal tract, and genital pathology both in WT and IL-17KO mice. WT C57BL/6 or IL-17 -/- OVX mice were implanted with E2 or placebo pellets. Both groups were intranasally immunized with a single dose of TK- HSV-2 at 10^4^ pfu/mouse. Six weeks later, both groups were challenged with WT HSV-2 at 10^4^ pfu/mouse as depicted in **Figure 1A**. Mice were monitored for genital pathology for 5 days post-challenge. Serum samples and vaginal washes were collected at day 5 post-challenge to assess HSV-2-specific total IgGs by ELISA and endpoint titers were determined. **(A)** HSV-2-specific total IgGs in serum samples and vag washes collected from WT OVX mice; **(B)** HSV-2-specific total IgGs in serum and vag washes collected from IL-17-/- OVX mice. **(C)** Genital pathology scores in E2 and placebo groups of WT mice, **(D)** Genital pathology scores in E2 and placebo IL-17 -/- mice. For **(A, B)**, data are representative of three independent experiments with similar results, and the bars indicate mean ± SEM. Data shown for **(C, D)** are representative of two independent experiments showing similar results. Data for panels **(A, B)** were analyzed by the unpaired, two-tailed *t* test. *, P<0.05.

Supplementary Figure 2 Serum and vaginal washes from E2-treated TK- HSV-2 immunized mice challenged with HSV-2 show pronounced inhibition of viral infection in Vero cells compared to placebo mice in an *in vitro* antibody neutralization assay using GFP-HSV-2 virus. WT C57BL/6 OVX mice were implanted with E2 or placebo pellets. Both groups were i.n. immunized with a single dose of TK- HSV-2 at 10^4^ pfu/mouse. Six weeks later, both groups were challenged with WT HSV-2 at 10^4^ pfu/mouse as depicted in **Figure 1A**. Serum samples and vaginal washes were collected at day 5 post-challenge. Serum and vaginal washes were assessed for their antibody-mediated neutralizing activity in an *in vitro* neutralization assay in Vero cells using GFP-HSV-2 virus. Sixty microliters (60 ul) of serum or vaginal wash samples were incubated with 60 ul of GFP-HSV-2 virus for 1 hour at 37°C (MOI of 0.5 to Vero cells). Then the pre-incubated virus and samples suspensions were added to confluent monolayers of Vero cells in 96-well plate and incubated at 37°C for 2 hours with intermittent swirling of the plate to allow virus to infect cells evenly and plate was then read on a CYTATION 7 imaging reader to determine GFP fluorescence at 395 nm/509 nm excitations/emissions wavelengths. **(A)** Representative GFP fluorescence that indicates the presence of HSV-2 virus are shown for both serum and vaginal washes collected from E2 treated and placebo mice. **(B)** Bar graphs showing the number of GFP+ Vero cells infected with GFP-HSV-2 virus as compared E2 treated serum and vaginal samples to their placebo counterparts. Data shown in panel B are representative of the three independent experiments performed under the same conditions showing reproducible results (n=6 animals/group). Graphs indicate mean ± SEM. Results were analyzed using the unpaired, two-tailed *t* test. **, P<0.01 ***; P<0.001.

Supplementary Figure 3 E2-treated mice show similar B cell profile in FGT compared to their placebo counterpart following intravaginal challenge with WT-HSV-2 in the absence of IL-17. IL-17-/- OVX mice were implanted with E2 or placebo pellets. Both groups were intranasally immunized with TK- HSV-2 at 10^4^ pfu/mouse. Six weeks later, both groups were challenged with WT-HSV-2 at 10^4^ pfu/mouse. Vaginal tissues were collected at day 5 post-challenge, pooled, processed and mononuclear cells were analyzed by flow cytometry. **(A)** Dot plot represents the proportion of B220+ and B220-CD138+ cells, **(B)** Bar graphs show mean frequencies and total numbers of B220+ B cells that were gated out of total live lymphocytes, and **(C)** Graphs display mean frequencies of B220-CD138+ plasma cells and their proportion was compared between E2-treated and placebo mice (n=5 animals/group). Data were analyzed by unpaired two-tailed *t* test. This experiment was conducted once.

Supplementary Figure 4 E2 treatment does not enhance the formation of memory B cells within the peripheral secondary lymphoid tissues in immunologically naive C57BL/6 OVX mice. WT C57BL/6 OVX mice were implanted with E2 or placebo pellets. Six weeks later, nasal associated lymphoid tissue (NALT), cervical lymph nodes (cLN), iliac lymph nodes (iLN) and spleen were collected, pooled, processed and mononuclear cells analyzed by flow cytometry. Memory B cells were gated as CD19+IgD- cells and identified by the heterogenous expression of CD73, CD80, or PD-L2. The differences in percentages of individual subsets (left panels) and the combined frequency of all subsets (right panels) was examined as displayed in **(A)** NALT, **(B)** cervical lymph nodes, **(C)** iliac lymph nodes, and **(D)** spleen. Data shown are collected from two independent experiments showing similar results. NALT tissues were pooled to 3 samples (n=3), cLN tissues to 4 samples (n=4) from n=6 mice/group; iLN (n=5) and spleen (n=5). Graphs represent mean ± SEM. Data for all left panels were analyzed using two-way ANOVA and for right panels by unpaired, two-tailed *t* test.

Supplementary Figure 5 E2 treatment enhances the formation of memory B cells within the peripheral secondary lymphoid tissues and in the vaginal tract in C57BL/6 OVX mice following 4-weeks of immunization followed by ivag HSV-2 challenge. WT C57BL/6 OVX mice were implanted with E2 or placebo pellets. Mice were either intranasally immunized with a single dose of 10^4^ pfu/mouse of TK- HSV-2 or left unimmunized. Four weeks later mice were challenged ivag with WT HSV-2 and 5 days later nasal associated lymphoid tissue (NALT), cervical lymph nodes (cLN), iliac lymph nodes (iLN) and spleen were collected, pooled, processed and mononuclear cells analyzed by flow cytometry. Memory B cells were gated as CD19+IgD- cells and identified by the heterogenous expression of CD73, CD80, or PD-L2. The percentages of individual subsets (left panels) and the total numbers of MBC subsets (right panels) was examined as displayed in **(A)** iliac lymph nodes, and **(B)** spleen. Data shown in panels **(A, B)** are collected from two independent experiments. NALT tissues were pooled to 3 samples (n=3), cLN tissues to 4 samples (n=4) from n=6 mice/group; iLN (n=5) and spleen (n=5). **(C)** HSV-2 specific memory B cells identified as IgG+CD19+IgD- cells were assessed from vaginal mucosa between un-immunized and immunized mice treated with E2 or placebo pellets. Data shown is from an independent experiment (n=5–6 animals/group). Graphs represent mean ± SEM. Data for all panels **(A-C)** were analyzed by two-way ANOVA. *, P <0.05; **, P <0.01; ***, P<0.001; ****, P<0.0001.
