# Supplementary figures and images for: Estradiol enhances B cell humoral immune responses against genital herpes simplex virus type 2 in mice through an IL-17 dependant pathway
Source: Front Immunol. 2025 Dec 1;16:1691163. doi: 10.3389/fimmu.2025.1691163 (PMC12702753; doi:10.3389/fimmu.2025.1691163)

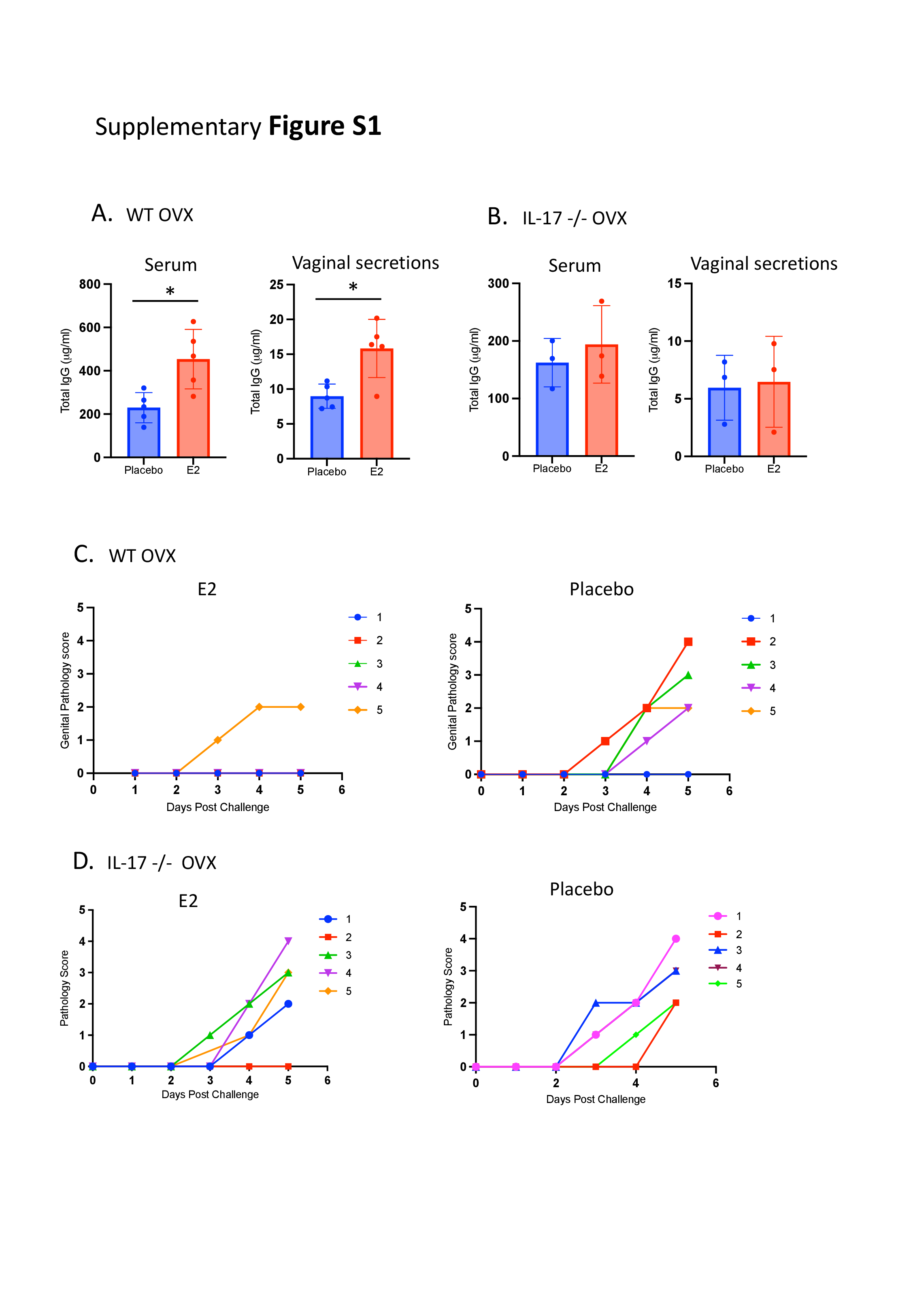

Supplement: Supplementary Table 1 — Characteristics of B cell subsets based on the expressions of surface markers. Naïve B cells are identified as B220+ and/or CD19+ live lymphocytes; Plasma cells were identified by gating B220-CD138+ cells among live cells. Memory B cells (MBC) are determined as CD19+ IgD- live lymphocytes and each Memory B cell subset was identified by gating CD73+, CD80+ and PD-L2+ among CD19+IgD- live B cells. [file Image1.tiff]

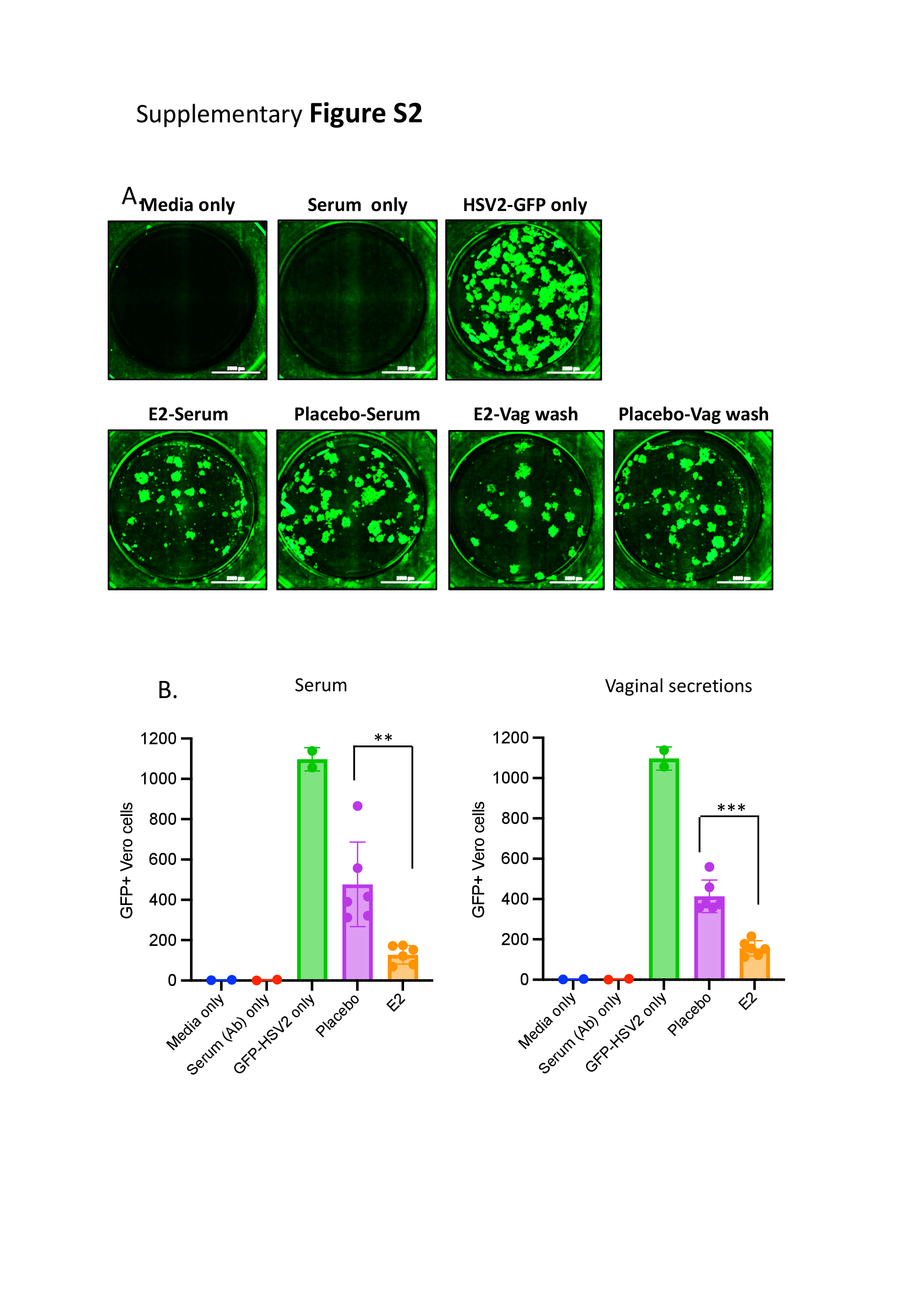

Supplement: Supplementary Figure 1 — Effects of E2 treatment on immunized mice challenged with HSV-2 on IgGs production in serum and vaginal tract, and genital pathology both in WT and IL-17KO mice. WT C57BL/6 or IL-17 -/- OVX mice were implanted with E2 or placebo pellets. Both groups were intranasally immunized with a single dose of TK- HSV-2 at 104 pfu/mouse. Six weeks later, both groups were challenged with WT HSV-2 at 104 pfu/mouse as depicted in Figure 1A. Mice were monitored for genital pathology for 5 days post-challenge. Serum samples and vaginal washes were collected at day 5 post-challenge to assess HSV-2-specific total IgGs by ELISA and endpoint titers were determined. (A) HSV-2-specific total IgGs in serum samples and vag washes collected from WT OVX mice; (B) HSV-2-specific total IgGs in serum and vag washes collected from IL-17-/- OVX mice. (C) Genital pathology scores in E2 and placebo groups of WT mice, (D) Genital pathology scores in E2 and placebo IL-17 -/- mice. For (A, B), data are representative of three independent experiments with similar results, and the bars indicate mean ± SEM. Data shown for (C, D) are representative of two independent experiments showing similar results. Data for panels (A, B) were analyzed by the unpaired, two-tailed t test. *, P<0.05. [file Image2.tiff]

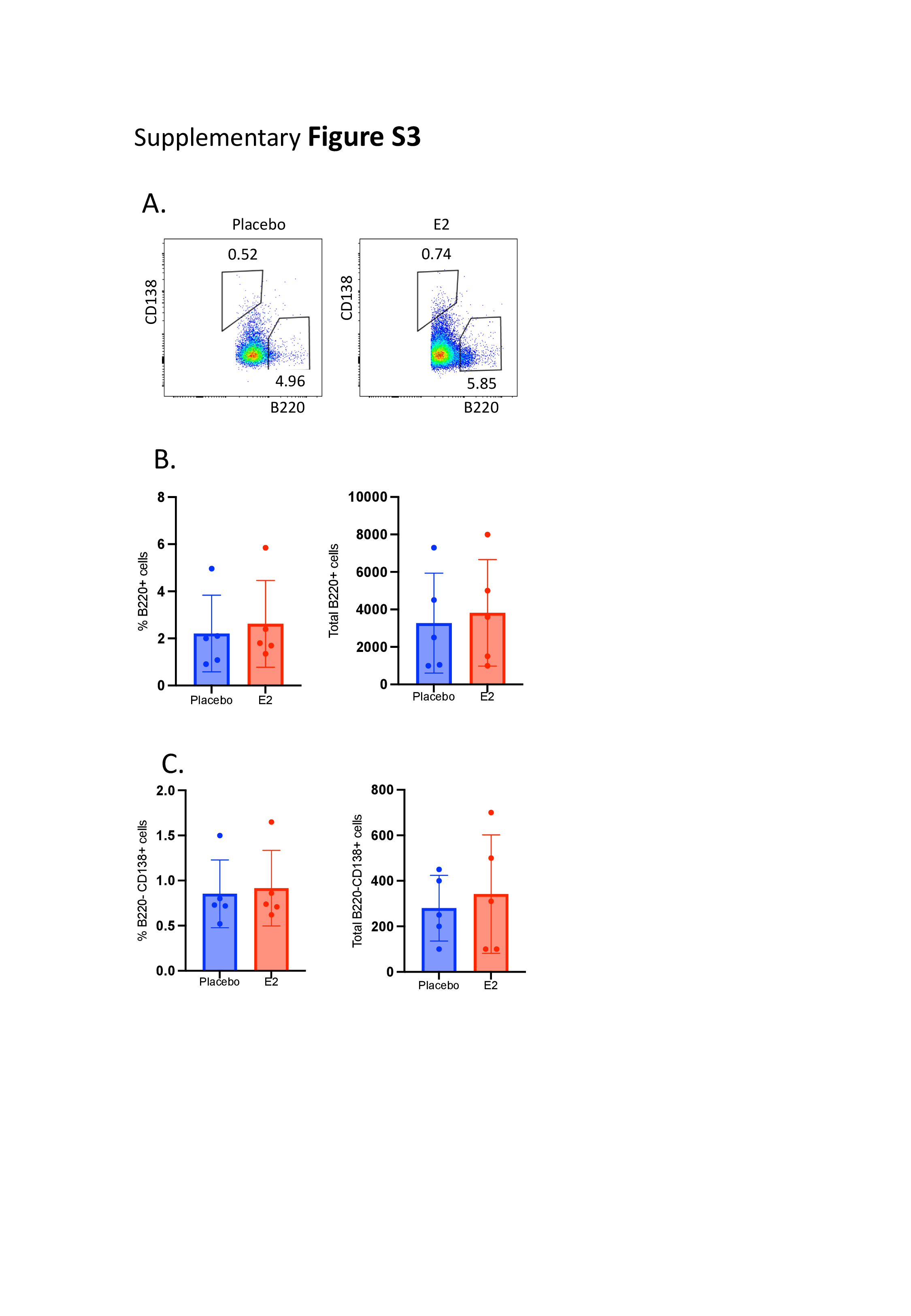

Supplement: Supplementary Figure 2 — Serum and vaginal washes from E2-treated TK- HSV-2 immunized mice challenged with HSV-2 show pronounced inhibition of viral infection in Vero cells compared to placebo mice in an in vitro antibody neutralization assay using GFP-HSV-2 virus. WT C57BL/6 OVX mice were implanted with E2 or placebo pellets. Both groups were i.n. immunized with a single dose of TK- HSV-2 at 104 pfu/mouse. Six weeks later, both groups were challenged with WT HSV-2 at 104 pfu/mouse as depicted in Figure 1A. Serum samples and vaginal washes were collected at day 5 post-challenge. Serum and vaginal washes were assessed for their antibody-mediated neutralizing activity in an in vitro neutralization assay in Vero cells using GFP-HSV-2 virus. Sixty microliters (60 ul) of serum or vaginal wash samples were incubated with 60 ul of GFP-HSV-2 virus for 1 hour at 37°C (MOI of 0.5 to Vero cells). Then the pre-incubated virus and samples suspensions were added to confluent monolayers of Vero cells in 96-well plate and incubated at 37°C for 2 hours with intermittent swirling of the plate to allow virus to infect cells evenly and plate was then read on a CYTATION 7 imaging reader to determine GFP fluorescence at 395 nm/509 nm excitations/emissions wavelengths. (A) Representative GFP fluorescence that indicates the presence of HSV-2 virus are shown for both serum and vaginal washes collected from E2 treated and placebo mice. (B) Bar graphs showing the number of GFP+ Vero cells infected with GFP-HSV-2 virus as compared E2 treated serum and vaginal samples to their placebo counterparts. Data shown in panel B are representative of the three independent experiments performed under the same conditions showing reproducible results (n=6 animals/group). Graphs indicate mean ± SEM. Results were analyzed using the unpaired, two-tailed t test. **, P<0.01 ***; P<0.001. [file Image3.tiff]

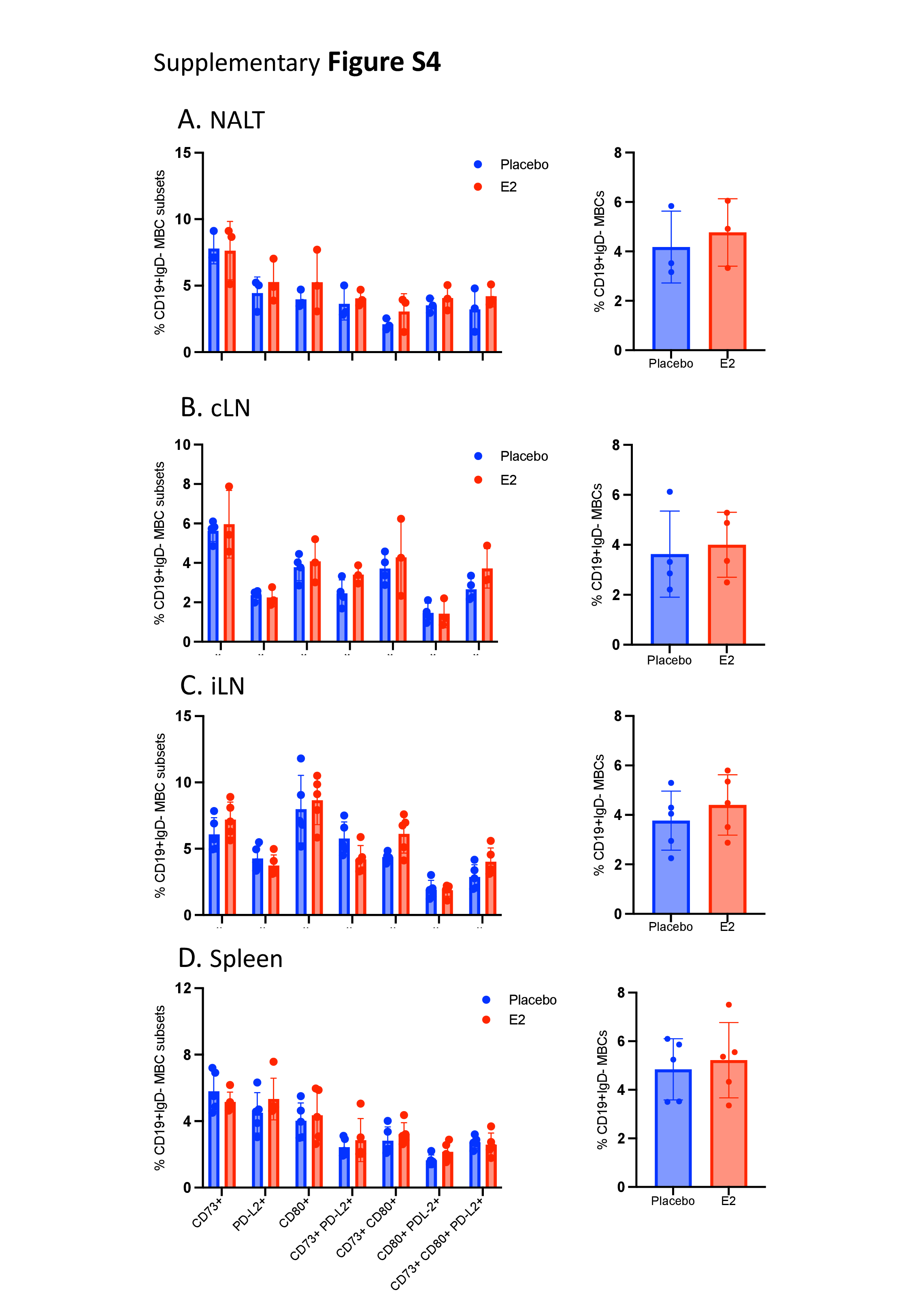

Supplement: Supplementary Figure 3 — E2-treated mice show similar B cell profile in FGT compared to their placebo counterpart following intravaginal challenge with WT-HSV-2 in the absence of IL-17. IL-17-/- OVX mice were implanted with E2 or placebo pellets. Both groups were intranasally immunized with TK- HSV-2 at 104 pfu/mouse. Six weeks later, both groups were challenged with WT-HSV-2 at 104 pfu/mouse. Vaginal tissues were collected at day 5 post-challenge, pooled, processed and mononuclear cells were analyzed by flow cytometry. (A) Dot plot represents the proportion of B220+ and B220-CD138+ cells, (B) Bar graphs show mean frequencies and total numbers of B220+ B cells that were gated out of total live lymphocytes, and (C) Graphs display mean frequencies of B220-CD138+ plasma cells and their proportion was compared between E2-treated and placebo mice (n=5 animals/group). Data were analyzed by unpaired two-tailed t test. This experiment was conducted once. [file Image4.tiff]

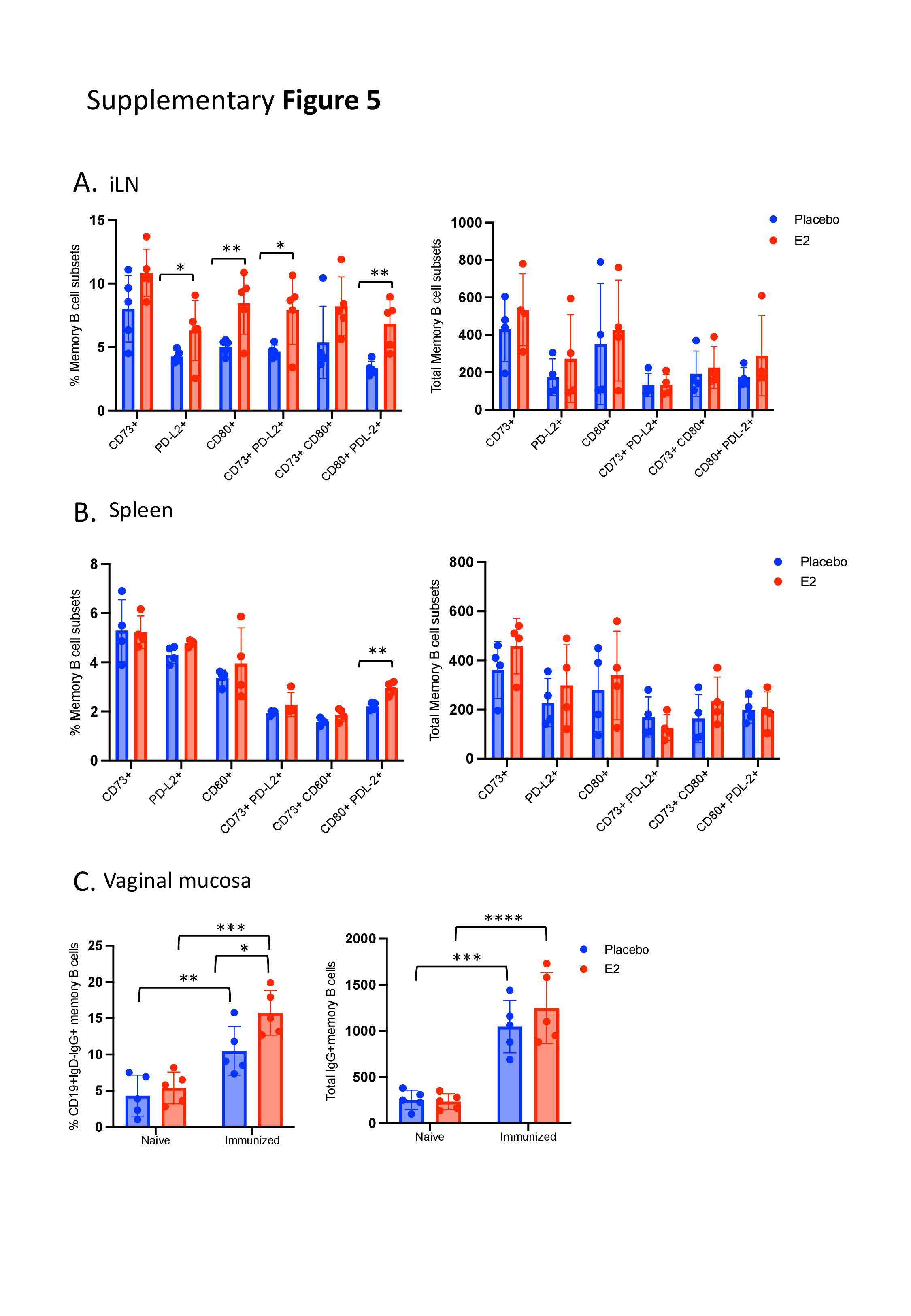

Supplement: Supplementary Figure 4 — E2 treatment does not enhance the formation of memory B cells within the peripheral secondary lymphoid tissues in immunologically naive C57BL/6 OVX mice. WT C57BL/6 OVX mice were implanted with E2 or placebo pellets. Six weeks later, nasal associated lymphoid tissue (NALT), cervical lymph nodes (cLN), iliac lymph nodes (iLN) and spleen were collected, pooled, processed and mononuclear cells analyzed by flow cytometry. Memory B cells were gated as CD19+IgD- cells and identified by the heterogenous expression of CD73, CD80, or PD-L2. The differences in percentages of individual subsets (left panels) and the combined frequency of all subsets (right panels) was examined as displayed in (A) NALT, (B) cervical lymph nodes, (C) iliac lymph nodes, and (D) spleen. Data shown are collected from two independent experiments showing similar results. NALT tissues were pooled to 3 samples (n=3), cLN tissues to 4 samples (n=4) from n=6 mice/group; iLN (n=5) and spleen (n=5). Graphs represent mean ± SEM. Data for all left panels were analyzed using two-way ANOVA and for right panels by unpaired, two-tailed t test. [file Image5.tiff]
